# In situ crystallographic mapping constrains sulfate precipitation and timing in Jezero crater, Mars

**DOI:** 10.1126/sciadv.adt3048

**Published:** 2025-04-16

**Authors:** Michael W. M. Jones, David T. Flannery, Joel A. Hurowitz, Michael M. Tice, Christoph E. Schrank, Abigail C. Allwood, Nicholas J. Tosca, David C. Catling, Scott J. VanBommel, Abigail L. Knight, Briana Ganly, Kirsten L. Siebach, Kathleen C. Benison, Adrian P. Broz, Maria-Paz Zorzano, Chris M. Heirwegh, Brendan J. Orenstein, Benton C. Clark, Kimberly P. Sinclair, Andrew O. Shumway, Lawrence A. Wade, Scott Davidoff, Peter Nemere, Austin P. Wright, Adrian E. Galvin, Nicholas Randazzo, Jesús Martinez-Frias, Lauren P. O’Neil

**Affiliations:** ^1^ Central Analytical Research Facility, Queensland University of Technology, Brisbane, 4000, Australia.; ^2^ School of Chemistry and Physics, Queensland University of Technology, Brisbane, 4000, Australia.; ^3^ Planetary Surface Exploration Group, Queensland University of Technology, Brisbane, 4000, Australia.; ^4^ School of Earth and Atmospheric Sciences, Queensland University of Technology, Brisbane, 4000, Australia.; ^5^ Department of Geosciences, Stony Brook University, Stony Brook, NY 11794, USA.; ^6^ Department of Geology and Geophysics, Texas A&M University, College Station, TX 77843, USA.; ^7^ Jet Propulsion Laboratory, California Institute of Technology, Pasadena, CA 91109, USA.; ^8^ Department of Earth Sciences, University of Cambridge, Cambridge, UK.; ^9^ Department of Earth and Space Sciences, University of Washington, Seattle WA 98195, USA.; ^10^ Department of Earth, Environmental, and Planetary Sciences, Washington University in St. Louis, St. Louis, MO, 63130, USA.; ^11^ Mineral Resources, Commonwealth Scientific and Industrial Research Organisation, Sydney, NSW, Australia.; ^12^ Department of Earth, Environmental and Planetary Sciences, Rice University, Houston, TX 77005, USA.; ^13^ Department of Geology and Geography, West Virginia University, Morgantown, WV 26506, USA.; ^14^ Department of Earth, Atmospheric and Planetary Sciences, Purdue University, West Lafayette, IN 47907, USA.; ^15^ Centro de Astrobiología (CAB), CSIC-INTA, 28850 Torrejón de Ardoz, Madrid, Spain.; ^16^ Space Science Institute, Boulder, CO 80301, USA.; ^17^ School of Computational Science and Engineering, Georgia Institute of Technology, Atlanta, GA 30332, USA.; ^18^ Department of Earth and Atmospheric Sciences, University of Alberta, Edmonton, Alberta, Canada.; ^19^ Institute of Geosciences, CSIC-UCM, Madrid, Spain.

## Abstract

Late-stage Ca-sulfate–filled fractures are common on Mars. Notably, the Shenandoah formation in the western edge of Jezero crater preserves a variety of Ca-sulfate minerals in the fine-grained siliciclastic rocks explored by the Perseverance rover. However, the depositional environment and timing of the formation of these sulfates are unknown. To address this outstanding problem, we developed a technique to map the crystal orientations of these sulfates in situ at two stratigraphically similar locations in the Shenandoah formation, allowing us to constrain the burial depth and paleoenvironment at the time of their precipitation. Our crystal orientation mapping results and outcrop-scale fracture analyses reveal two different generations of Ca-sulfates: one likely precipitated in the shallow subsurface and a second one that formed at a burial depth below 80 meters. These results indicate that two studied locations capture two different times and distinct chemical conditions in the sedimentary history of the Shenandoah formation, providing multiple opportunities to evaluate surface and subsurface habitability.

## Teaser

Crystal orientation analysis of Ca-sulfates on Mars indicates precipitation at varying times covering multiple paleoenvironments.

## INTRODUCTION

Since landing in Jezero crater on Mars in February 2021, the Mars 2020 Perseverance rover has explored igneous rocks exposed in the crater floor (*1*, *2*) and sedimentary rocks preserved in paleolake and deltaic environments (*3*). The overarching goals of the Mars 2020 mission are to constrain the habitability of these paleoenvironments, to search for biosignatures (*4*) and to collect a compelling cache of samples for potential Mars Sample Return (*5*). Fine-grained clastic rocks were analyzed by Perseverance as the rover traversed the main sedimentary fan exposed in the western edge of Jezero crater, in a succession informally known as the “Shenandoah formation” (fig. S1) (*6*). Two study areas in this region, at “Hogwallow Flats” (“Wildcat Ridge” outcrop) and at “Yori Pass” (“Hidden Harbor” outcrop), are notable for abundant sulfate minerals present in clasts, cements, vugs, and in veins (*7*, *8*).

Sulfates are common on Mars, occurring in soils at typically 4 to 8 wt % budgeted as SO_3_ (*9*, *10*), in regional light-toned layered deposits (*11*, *12*), as gypsum (CaSO_4_·2H_2_O) cements in siltstones and sandstones (*13*), and as calcium sulfate veins at the rover sites of Opportunity (*14*), Curiosity (*15*, *16*), and Perseverance (described here). Most rock units encountered by Curiosity contain calcium sulfate veins, which reveal intriguing aspects of the geological history. For example, bassanite (CaSO_4_·0.5H_2_O) veins, perhaps once gypsum (*17*), cutting the Yellowknife Bay formation (*18*) and complex vein networks in the Murray formation (*19*) attest to multiple episodes of postdepositional fluid flow and hydrofracturing. Given their ubiquity, sulfate-precipitating fluids must have played an important role in water and element transfer on Mars. Therefore, determining the exact composition and origin of sulfate veins can provide insights into Mars’ hydrological history.

At Hogwallow Flats and Yori Pass in the Shenandoah formation, Perseverance’s Sample Caching System (*20*) collected three rock cores for potential return to Earth: “Hazeltop” and “Bearwallow” at Wildcat Ridge and “Kukaklek” at Hidden Harbor (figs. S1 and S2; see Table 1 for corresponding names). These sulfates offer remarkable opportunities to investigate past environmental conditions and potentially also provide evidence of past life on Mars (*8*, *21*). However, the timing and context of the formation of these minerals are crucial to interpreting such evidence.

**Table 1.** Target names. Member, outcrop, abrasion patch, and core names for the two studied workspaces at the Shenandoah formation.

Member	Outcrop	Abrasion patch	Core(s)
Hogwallow Flats	Wildcat Ridge (WR)	Berry Hollow (BH)	Hazeltop, Bearwallow
Yori Pass	Hidden Harbor (HH)	Uganik Island (UI)	Kukaklek

At both Hogwallow Flats and Yori Pass, the Shenandoah formation is composed of fine-grained, sulfate-cemented, planar-laminated, and low-angle cross-stratified sandstone and siltstone intercalated with recessive and resistant, meter-scale beds preserving soft-sediment deformation structures (*6*). At Hogwallow Flats, the Shenandoah formation (fm). dips 5° to 8° to the southeast (*22*). On the basis of similar orientations and elevations and shared sedimentological and lithological characteristics, the Hidden Harbor outcrop (Yori Pass member) is likely the lateral equivalent of the Wildcat Ridge outcrop (Hogwallow Flats member) (*6*). SuperCam (SCAM) detected sulfates at the Wildcat Ridge and Hidden Harbor outcrops in infrared (IR) spectra (*8*). The absence of hydration bands between 3200 and 3700 cm^−1^ in the Raman spectra indicates the presence of anhydrite (CaSO_4_) (*23*).

**Fig. 1.**
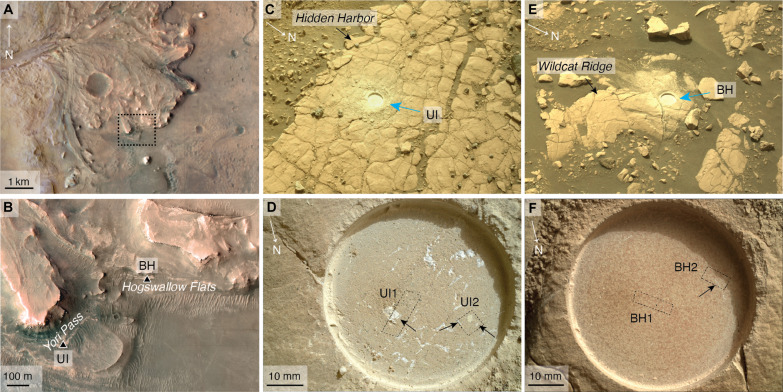
Location of the two abrasion patches at the Western Jezero fan. (**A**) [zoomed in: (**B**)] Locations of UI and the BH abrasion patches. The location of each abrasion patch on each outcrop is shown in (**C**) and (**D**) and (**E**) and (**F**) for UI and BH, respectively. Blue arrows in (C) and (E) indicate the location of each patch, and the outcrop-scale fracture network can be easily seen in each case. Dashed boxes in (D) and (E) show the scan footprints for UI1, UI2, BH1, and BH2, and black arrows in (D) and (F) highlight CaSO_4_ regions within the PIXL scan footprints. No substantial CaSO_4_ features were observed by PIXL in BH1. Images in (C) and (E) were generated by Navcam, and images in (D) and (F) were generated by WATSON.

Abrasion patches (*20*), informally named Berry Hollow (BH) and Uganik Island (UI), were created by Perseverance on mission sol 504 and 612 at the Hogwallow Flats and Yori Pass members, respectively (Fig. 1). SHERLOC (Scanning Habitable Environments with Raman and Luminescence for Organics and Chemicals) analyses of the light-toned veins and vugs at the BH and UI abrasion patches confirm the presence of anhydrite with some hydrated phases also detected, likely hydrated Mg sulfates (*24*). In addition, SCAM IR analysis indicates the presence of anhydrite at UI (*8*). These light-toned veins and vugs and the surrounding host rock were targeted by the Planetary Instrument of X-ray Lithochemistry [PIXL; (*25*)], one of the proximity science instruments on the arm of Perseverance (*4*), on sols 505 (BH1) and 507 (BH2) and 614 (UI1) and 617 (UI2) (Fig. 1, C to F). In these scans, PIXL generated quantitative elemental maps (*25*) and detected diffraction peaks (*2*, *26*, *27*). Diffraction peaks detected in a PIXL spectrum represent elastically backscattered photons, which can be mapped with a technique called x-ray backscatter diffraction mapping (XBDM) (*28*). XBDM is equivalent to electron backscatter diffraction microscopy (EBSD) (*29*) and serves to produce maps of crystal orientation. In contrast to EBSD, XBDM does not require a polished coated surface (*28*), making it ideally applicable to in situ studies on Mars. Here, we extend the XBDM method by using a combination of fluorescence and diffraction information to uniquely identify stoichiometrically similar minerals (*30*) and determine their crystallographic orientations to match the functionality of EBSD more closely. These results, together with geometric and outcrop-scale fracture analyses, allow us to determine the formation mechanisms of these veins and vugs and therefore constrain the sulfate precipitation and diagenetic processes at Wildcat Ridge and Hidden Harbor.

## RESULTS

### Composition and mineral phases of the veins and vugs

**Fig. 2.**
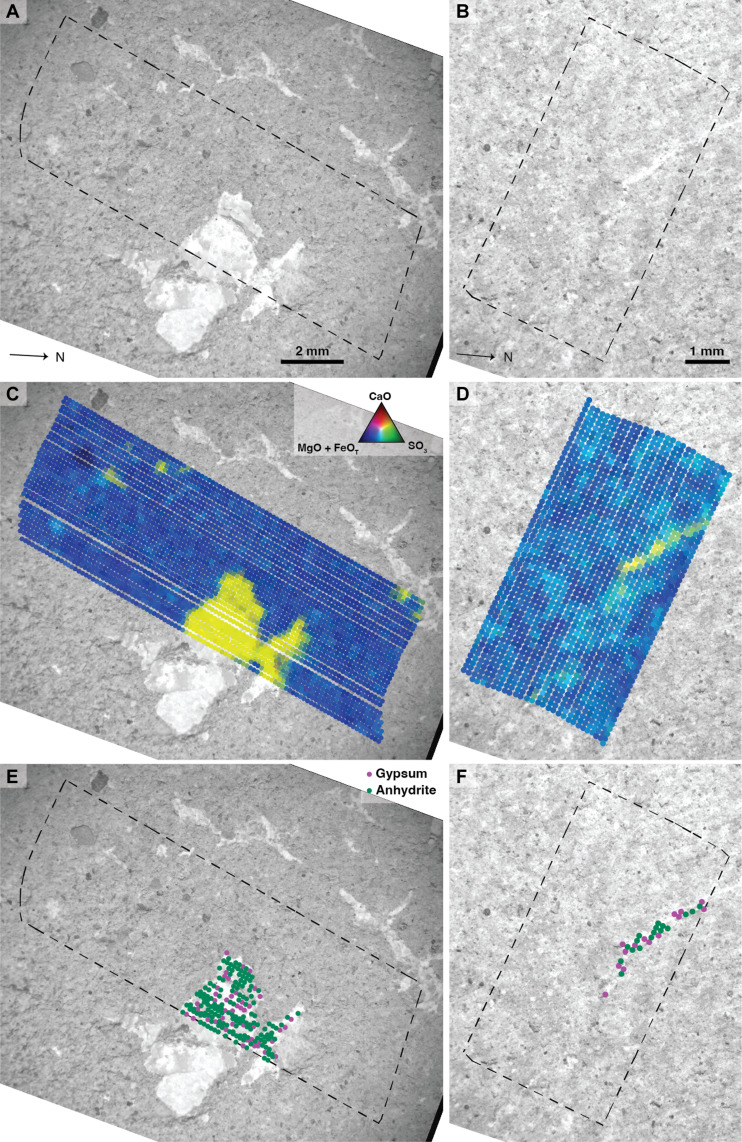
PIXL analysis indicating Ca-sulfate–rich regions in the two abrasion patches. Scan footprints for UI1 and BH2 (**A** and **B**) shown overlaid on SHERLOC ACI images. The scan locations are shown in Fig. 1 (D and F), respectively. Light toned regions are interpreted as CaSO4, which is confirmed by mapping CaO (max, 4.8; 7.9 mmol g^−1^), SO_3_ (max, 6.0; 8.3 mmol g^−1^), and FeO_T_ + MgO (max, 7.3; 7.7 mmol g^−1^), as three-color RGB images (**C** and **D**). CaSO_4_ minerals appear yellow according to the color mixing triangle in (C) [applies to (C) and (D)]. CaSO_4_ mineral identification maps for the two scans are shown in (**E**) and (**F**) and that anhydrite is the dominant phase. Comparison between the XRF-identified CaSO_4_ minerals [(C) and (D)] and the diffraction-based CaSO_4_ mineral identification [(E) and (F)] indicates that the majority of the CaSO_4_ minerals had sufficient diffraction to enable identification.

PIXL’s x-ray fluorescence (XRF) analysis of the scanned regions confirms that the light-toned material (visible in BH2, UI1, and UI2) is mainly composed of calcium and sulfur (reported as CaO + SO_3_ here) and is largely devoid of other measurable elements (e.g., <10 wt % total other measured elements for the larger feature at UI1; Fig. 2 and figs. S3 to S5 and S7). The BH1 scan does not contain any CaO + SO_3_ regions and is excluded from further analysis (fig. S6). The host rock is rich in FeO_T_, MgO, and SiO_2_ but is largely free of CaO and lower in SO_3_ compared to the CaO + SO_3_–rich regions (see figs. S3 to S5). Diffraction from the light-toned material in both scans was compared to a database (*31*, *32*) of possible diffraction spectra detected by PIXL for all orientations of anhydrite (*33*), gypsum (*34*), and bassanite (*35*). Indexing was successful for 80% of all beam locations, with the remaining 20% being statistically ambiguous (no clear mineral able to be identified at a *P* value of less than 0.05) and therefore not included (see the Supplementary Materials).

Exposing fully hydrated calcium sulfate (i.e., gypsum) to the martian atmosphere may result in conversion to the partially dehydrated form (i.e., bassanite). Mars Environmental Dynamics Analyzer (MEDA) observations suggest that the daytime environmental conditions give rise to the possibility that CaSO_4_·2H_2_O could be dehydrated during the day and rehydrated at night at temperatures below 195 K (*36*). However, before abrasion, sulfates remain stable in several different hydration states due to the slow kinetics of the process and the protection of the overlying rock. The analysis of sieved (<150 μm) sulfates at Gale crater shows that minimal conversion is likely to occur after 4 sols, while significant conversion is observed after 8 sols (*13*). From the time of the abrasion, BH1 and BH2 were measured within 1 and 3 sols, respectively, and UI1 and UI2 were measured within 2 and 5 sols. The proportions of different Ca-sulfate dehydration phases in BH2 and UI1 are approximately 37 and 20% gypsum, 45 and 66% anhydrite, and 18 and 14% bassanite, respectively. UI2 contains similar proportions of calcium sulfates as UI1 (fig. S8), implying that no conversion had occurred between abrasion and data collection. Because many bassanite diffraction peaks overlap with those of gypsum and anhydrite, bassanite identification is unreliable (*13*), and these beam locations were removed from further analysis. Beam locations identified as gypsum and anhydrite (Fig. 2, E and F) show no clear spatial grouping. BH had a greater proportion of gypsum relative to anhydrite than UI, suggesting that the fluids that precipitated Ca-sulfate at BH had a higher water activity or lower temperature (*37*–*39*).

### Outcrop-scale fracture network

**Fig. 3.**
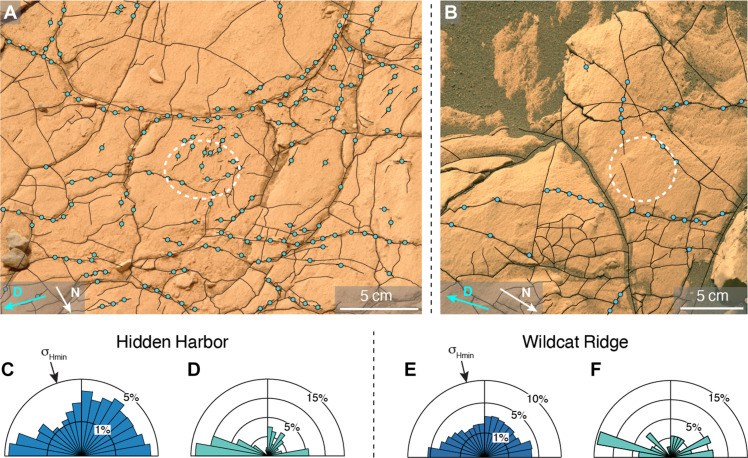
Fracture analysis of the studied outcrops. Mastcam-Z photographs of the decimeter-scale fracture networks at (**A**) Hidden Harbor (HH) and (**B**) Wildcat Ridge (WR). Black solid lines mark fracture traces, while the blue circles indicate locations where sulfate veins are exposed. The dashed white ellipses in (A) and (B) indicate the location of the UI and BH abrasion patches, respectively. (**C** and **D**) Relative frequency orientation distribution of fracture traces and sulfate vein segments at HH respectively visualized as polar histograms. (**E** and **F**) Equivalent data for WR. The black arrow at the histogram perimeter in (C) and (E) indicates the σ_Hmin_ (*42*) orientation of ~10°/190° NS at HH and ~45°/225° NE/SW at WR. This direction aligns with the orientations derived from abrasion patch analyses (see Figs. 4 and 5). White and cyan arrows in (A) and (B) show the direction of martian North “N” and the downhill direction “D” and apply to the relevant polar histograms.

The outcrop-scale fracture network at Hidden Harbor (Figs. 1C and 3) and Wildcat Ridge (Figs. 1E and 3) is well connected and polygonal, with a mean fracture spacing of 1.4 and 2.2 cm, respectively, measured with the intercept method (*40*). The orientation frequency distribution of all fracture traces mapped within the horizontal outcrop plane displays two weak, mutually perpendicular modes of the orientation distribution at 295° and 205° (Hidden Harbor; Figs. 1C and 3A) 153° and 253° (Wildcat Ridge; Figs. 1E and 3B). Oblique outcrop photographs show boulders and steep edges of the rocky outcrop with mainly vertical fracture traces and some bedding-parallel fractures. Hence, most fractures are likely subvertical. In addition, there is no evidence for shear displacement along fractures. Therefore, the outcrop-scale fracture system at both outcrops likely represents subvertical extension fractures (*41*).

The frequency distribution of the orientation of exposed vein segments at Hidden Harbor resembles that of the entire fracture network, i.e., fractures with and without visible mineral infill, but with a much more pronounced mode at 295°. At Wildcat Ridge, sulfates mainly occur in eight separate decimeter-scale veins (Fig. 3B). As a result, the orientation frequency distribution of exposed vein segments is much less isotropic than that of the wider fracture network, with the strongest mode at 163° and two smaller modes at 323° and 303°. Both orientation frequency distributions (i.e., of the entire fracture network and the visible veins only) at Hidden Harbor show a marked minimum for ~N-S-striking fracture traces. By estimating the orientation of the minimum principal stress within the outcrop plane (σ_Hmin_) that maximizes the dilation tendency on the entire fracture network (*42*), we derive an σ_Hmin_ trending ~10°/190° (NS; Fig. 3) at Hidden Harbor, supported by the relative scarcity of N-S-striking fracture traces. Similarly, maximizing the dilation tendency on the entire fracture network at Wildcat Ridge in the horizontal plane gives a trend of ~45°/225° for σ_Hmin_ (NE/SW; Fig. 3), which is approximately orthogonal to the most common strike of the exposed vein segments.

The polygonal fracture networks formed in a stress field where the maximum principal stress σ_1_ was vertical, most likely imposed by the overburden of the sediment or sedimentary rock present at the time of fracture formation (*43*), similar to that derived from 3D exposures of sulfate veins at Gale crater (*44*). However, in contrast to the fractures observed at Gale crater, the presence of a preferred orientation of exposed vein segments suggests that there was a small difference in magnitude of the horizontal stresses (intermediate and minimum principal stresses σ_2_ and σ_3_, respectively).

### UI abrasion patch veins

**Fig. 4.**
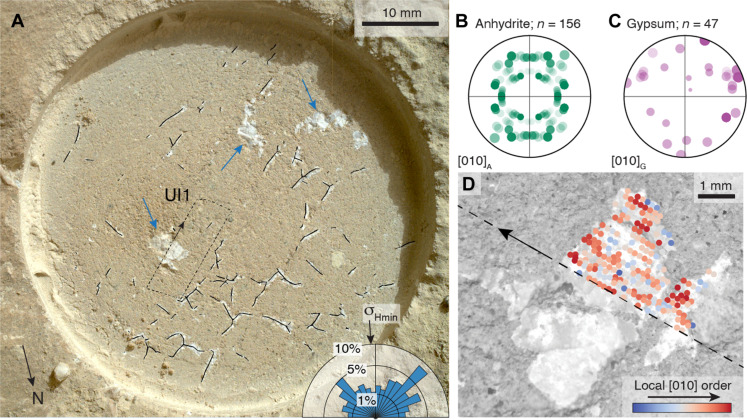
UI abrasion patch analysis. SHERLOC WATSON image of the UI abrasion patch (**A**) showing sulfate-filled fractures (white regions) overlaid with a binary image of fracture traces. The blue arrows mark polygonal vugs with low aspect ratio omitted in the orientation analysis. Polar histogram of the orientation of fracture traces is shown in the lower right, indicating a σ_Hmin_ direction of ~10°/190° NS. Random [010] pole figure for anhydrite (**B**) and gypsum (**C**) at UI2 [dashed box in (A)] indicates no Crystallographic Preferred Orientation (CPO). Mapping the regional crystallographic alignment of the [010] plane for both anhydrite and gypsum (**D**) reveals regions of high regional alignment throughout the area, suggesting a blocky texture. This blocky texture can also be seen in the sulfate mass immediately to the lower left of the mapped region. Black arrows in (A) and (D) show relative orientation between abrasion patch and scan.

The sulfate veins in the UI abrasion patch at Hidden Harbor (Figs. 1D and 3A) have lengths between 0.4 and 8 mm and widths between 0.1 and 4 mm, as measured by the Feret’s minimum and maximum diameter (*45*). Their aspect ratios range from 1.2 to 8.7 with a mean of 3. The largest sulfate regions have low aspect ratios and approximately rectangular shape. Several larger cracks display the shape of three-armed stars (Fig. 4A). The veins and vugs appear largely isolated at the scale of observation. Omitting the low-aspect ratio vugs, the orientation frequency distribution of traces shows two modes at right angles to each other, namely, 240° and 150° (Fig. 4A), yielding σ_Hmin_ trending ~10°/190° (NS), in line with the outcrop analysis (Fig. 3A).

PIXL scanned the cement of the ~4-mm-scale “fish-shaped” vug (*7*) in the UI1 scan (Figs. 2A and 3A). The optical images reveal a blocky cement texture in this vug (Figs. 2A and 3A) (*7*). The pole figures for the [010] reflections of gypsum and anhydrite do not show a crystallographic preferred orientation for the overall texture pattern (Fig. 4, B and C) (*46*). However, mapping the regional crystallographic alignment of the [010] pole (Fig. 4D) for gypsum and anhydrite shows discrete regions of high regional alignment (see the Supplementary Materials), which are interpreted as individual crystals. This blocky crystal texture is not diagnostic of the fracture mechanism or fracture kinematics (*47*) and can be explained either by a fast nucleation rate during the growth of a primary vug cement in an open fluid-filled cavity or due to later static recrystallization or replacement of an earlier cement (*47*). The size of domains indicates regions of crystal alignment (as either a single crystal or multiple aligned smaller crystals) with a size on the order of several hundreds of micrometers, in line with the blocky cement texture identified previously (Figs. 2A and 3A) (*7*).

### BH abrasion patch vein

**Fig. 5.**
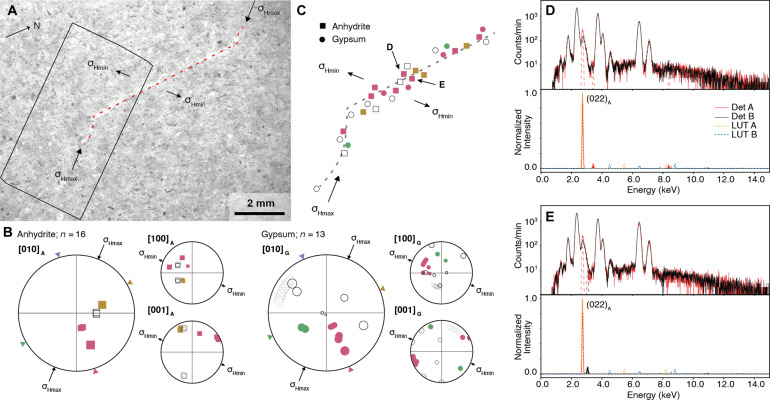
BH abrasion patch analysis. Visual inspection of the vein geometry at BH [(**A**), dashed line] permits identification of σ_Hmax_ and σ_Hmin_ directions with σ_Hmin_ of ~45°/225° (NE/SW). Beam locations identified as gypsum (circles) and anhydrite (squares) in Fig. 2E are shown in stereographic projections with combined hemispheres in (**B**). The colors of each beam location correspond to alignment of the [010] to the expected direction (shown as triangles on the outer perimeter of the stereo projections) for anhydrite (40° from σ_Hmax_) and gypsum (45° from σ_Hmax_) for the given principal stress direction σ_Hmax_. The [100] and [001] figures are oriented in the expected orthogonal planes to the [010] figure and provide additional constraints on crystal orientations. Open squares and circles depict results from beam spots where [010] planes are not aligned with respect to the vein geometry. Mapping the beam locations (**C**) shows high alignment of the [010] plane throughout the vein, confirming the stress field identified through visual inspection. PIXL spectra from two PMCs are presented in (**D**) and (**E**), where the prominent peak in the data [red dashed peak in upper plots of (D) and (E)] has an excellent correlation (cross-correlation coefficient = 0.98 in each case) to the anhydrite (022) peak from the look up table [LUT; orange dotted peak in lower plots at ~2.7 keV in (D) and (E)]. Other peaks in the data and LUT have negligible intensity.

The BH abrasion patch at Wildcat Ridge exposes one sulfate vein (Figs. 1F and 2B) with the shape of a classical wing crack (*48*, *49*). The vein has a straight ~4-mm–long NS-striking central segment flanked by ~1-mm–long curved, tapering termini, making a ~±35° angle with the central segment (Fig. 4A). The wing crack geometry implies that the vein propagated as mixed-mode crack with a left-lateral shear component in the outcrop plane. The orientation of the maximum and minimum principal stresses (σ_Hmax_ and σ_Hmin_, respectively) within the horizontal outcrop plane can be determined from the wing crack geometry (Figs. 2B and 5A) (*49*), giving a trend of ~45°/225° for σ_Hmin_ (NE/SW), the same as the outcrop-scale fracture network (Fig. 3). Crystal-texture analysis demonstrates that both anhydrite and gypsum crystals of this vein exhibit a crystallographic preferred orientation. Pole figures of individual beam spots containing anhydrite (squares; space group Cmcm) and gypsum (circles; space group C2/c) show that most [010] planes of both minerals strike approximately parallel to the outcrop trace of the wall of the main vein segment, and their poles make an angle of about 45° and 40° to the inferred σ_Hmax_ direction, respectively (Fig. 5) (*46*). The [010] plane of both minerals is inclined (Fig. 5B). Analysis of the fluorescence signal shows that the vein dips to the W at approximately 40° (fig. S9). The vein geometry, orientation, and crystal texture imply that the central vein segment experienced both an opening and shear displacement with either an elongate-blocky or stretching-vein fabric (*47*). Therefore, our observations suggest that the vein at BH probably formed as a mixed-mode crack-seal vein filled with primary Ca-sulfates.

Considering the parabolic nature of the brittle yield envelope for rocks in the tensile domain, this also means that this vein formed at greater differential stress [e.g., figure 3 in (*47*)], and thus greater burial depth, compared to the extension fractures observed at Wildcat Ridge and Hidden Harbor. It is possible that some of the larger fractures mapped at the surface of Wildcat Ridge (Fig. 3B) are also mixed-mode fractures with a dip angle of <90° and may have formed in a more consolidated rock at a greater burial depth than the vertical extension cracks observed elsewhere.

### Origin of the fracture network and state of stress

The outcrop-scale polygonal vertical extension fractures could have formed because of volumetric shrinkage of sediment at or near the surface [desiccation or syneresis, e.g., (*50*, *51*)] or by hydraulic fracturing in partially or fully lithified sediment with overpressured pore fluid (*43*). While there is no observable diagnostic feature that allows us to ascertain which of these mechanisms operated, rock mechanics theory suggests that the maximum burial depth is constrained to a differential stress of <4 *T* where *T* is the tensile strength of the rock (*41*). However, BH exposes a moderately dipping mixed-mode crack-seal vein, which formed at a differential stress of >4 *T* (*41*) and thus at greater burial depth than the polygonal, vertical extension fractures. For a rock with relatively low tensile strength (*52*), we obtain a burial depth of greater than 80 m during the formation of the BH wing crack (fig. S10).

## DISCUSSION

### Sulfate diagenetic history

Instruments on previous Mars missions have sometimes identified the hydration state of calcium sulfate. On Opportunity, Pancam IR reflectance at 1009 nm indicated a centimeter-wide gypsum vein at Endeavour Crater (*53*), while on Curiosity, a Mastcam IR reflectance spectrum slope at 937 to 1013 nm suggested gypsum in thicker veins at Yellowknife Bay (*13*). Hydrogen emission at 656 nm from ChemCam laser-induced breakdown spectroscopy was used to infer hydrated CaSO_4_ in veins (*18*), which are predominantly bassanite that may have once been gypsum (*17*). Crystallographic differences allowed distinction between gypsum, bassanite, and anhydrite for in situ vein samples analyzed by the x-ray diffraction instrument, CheMin (*13*, *17*). However, inferred burial depths for veins have generally been uncertain given the argument that hydrofracturing might occur at any depth at sufficiently high fluid pressures (*19*).

**Fig. 6.**
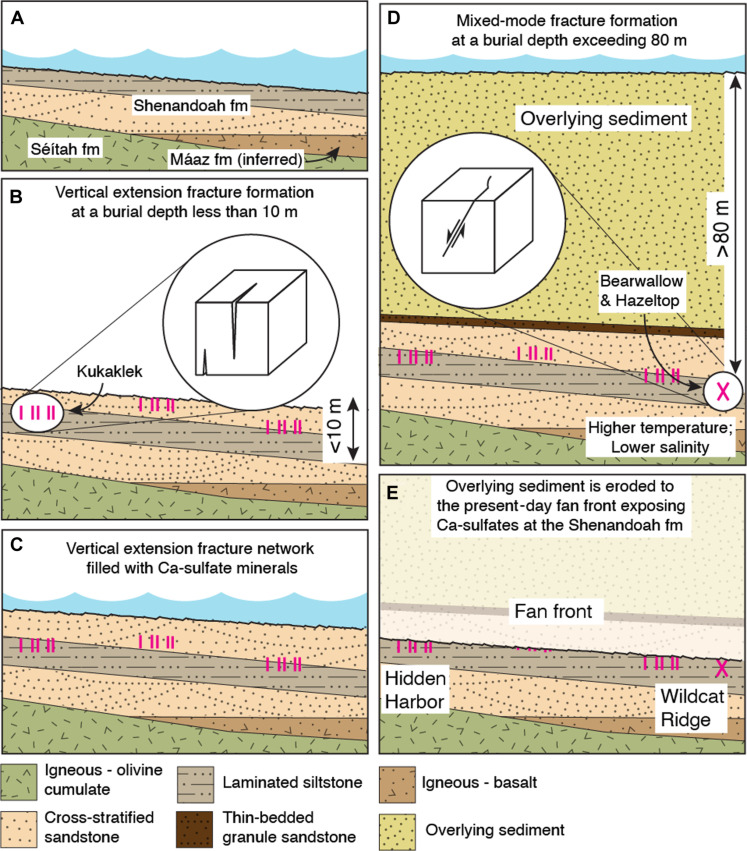
Deposition sequence. The sedimentary Shenandoah formation is deposited over the igneous Séítah and inferred Máaz formations (**A**) (*6*). Vertical extension fractures (vertical magenta lines) form in the shallow subsurface (**B**). These fractures are filled with primary or secondary Ca-sulfates (**C**). More than 80 m of additional material is deposited over the Shenandoah formation causing a mixed mode shear fracture (magenta X) to form (**D**) with primary Ca-sulfate precipitated. At this depth, the temperature is expected to be higher than at the surface (*57*). Therefore, the increased abundance of gypsum in the mixed mode shear fracture compared to the vertical extension fractures indicates a decreasing salinity over time. Excess material is eroded exposing a mixed-mode shear fracture at the BH abrasion patch at Wildcat Ridge and vertical extension fractures throughout the formation (**E**).

Analysis of the Ca-sulfate–filled fractures at the outcrops at Hidden Harbor and Wildcat Ridge and the UI abrasion reveals that they are vertical extension fractures that could have formed because of volumetric shrinkage of sediment at or near the surface [desiccation or syneresis, e.g., (*50*, *51*)] or by hydraulic fracturing in partially or fully lithified sediment with overpressured pore fluid (*43*) at depths as shallow as tens of meters (Fig. 6B). The analysis does not indicate if the observed Ca-sulfates were a primary or replacement cement. If they are a replacement, then thermodynamic constraints suggest that the original mineral(s) occupying these fractures may have included chloride minerals dominated by Na, K, Mg, and/or Ca, especially as SO_4_ and dissolved inorganic carbon were progressively removed from the fluid by precipitation of Fe/Mg-carbonate- and Fe/Mg-sulfate minerals (*54*), the latter of which are abundant outside of the Ca-sulfate filled regions in the UI abrasion. During diagenesis, these fractures could then have been preferentially replaced by Ca-sulfates as late-stage and highly soluble phases were dissolved and replaced by relatively insoluble gypsum and/or anhydrite.

In contrast, crystallographic and geometric analysis of the crack seal vein at the BH abrasion indicates formation at a minimum burial depth of ~80 m, with Ca-sulfate as a primary cement (Fig. 6C and fig. S10), at least twice the depth suggested previously (*8*, *55*). Assuming the sedimentary fan front once extended at least 4 km further E, and at least 3 km further SE to Santa Cruz and Pilot Pinnacle buttes, respectively (*56*), and has since been eroded back to its current position, the 80-m minimum depth estimate can be accommodated by the ~110 m elevation difference between BH and the top of the sedimentary fan. Compared to the features analyzed at UI, the BH Ca-sulfate has a higher proportion of gypsum. Differences in gypsum and anhydrite precipitation conditions can be related to variations in temperature and/or salinity (*37*–*39*), with gypsum favored at lower temperature and/or salinity. Since the gypsum-dominant crack-seal vein formed at greater depth, and therefore greater temperature (*57*) than the extension fractures elsewhere in the formation, it likely precipitated from a solution with lower salinity.

Despite the possibility of a replacive origin, Ca-sulfate in Kukaklek (from Hidden Harbor) contains abundant vein and vug cements likely formed in the shallow subsurface. In contrast, Ca-sulfate in the Hazeltop and Bearwallow samples (from Wildcat Ridge) may preserve information from a less saline environment deeper in the subsurface at a higher temperature. These samples therefore represent two different paleoenvironments, capturing a range of saline surface and groundwaters present at the Shenandoah formation providing two unique opportunities in the search for extraterrestrial biosignatures (*58*–*62*) on their potential return to Earth.

## MATERIALS AND METHODS

### PIXL data acquisition and processing

XRF data were collected by the PIXL (*25*). To create a map, PIXL is placed at a standoff distance of ~25.5 mm from the surface and scanned via a hexapod, with data collected with a 10-s dwell time in a regular grid with 120 μm spacing. Each data acquisition point is given a PIXL Motor Control (PMC) number for each scan. A Rh x-ray tube operating at 28 keV is used to generate x-rays with the incident beam focused to ~120 μm spot (at 8 keV) at the surface using a polycapillary optic (*25*). Because of the nature of the polycapillary optic, the focus size depends on the x-ray energy resulting in a smaller beam size at higher energies and vice versa (*26*, *63*). Excited XRF photons are collected with a pair of silicon drift detectors placed on either side of the optic (*25*).

Individual XRF spectra for each detector and PMC were quantified as oxides with PIQUANT (*64*) and visualized in PIXLISE (*65*). Elemental quantifications for each PMC were cleaned to remove effects due to surface topography and diffraction in PIXLISE (*66*). Data for MgO, Al_2_O_3_, SiO_2_, MnO, FeO_T_, SO_3_, CaO, and SrO were exported in units of millimoles per gram for the three-color images in Fig. 2 and the ternary diagrams in fig. S7 and weight % for the individual elemental images in figs. S3 to S6. For BH1, BH2, and UI2, SrO values are below the limit of detection due to low signal and overlap with the Fe pileup peak and are therefore not presented.

### Mapping crystallography with PIXL

The process for mapping crystallography with PIXL is as follows. First, XRF data were used to select PMCs that contained Ca-sulfate minerals. Second, we modeled the diffraction peaks that the PIXL would observe from the identified possible Ca-sulfate minerals (gypsum, bassanite, and anhydrite) to create a look up table (LUT), in a process called the “forward simulation.” Third, the observed diffraction was compared to this LUT, and the statistically most likely mineral was selected, along with its relative crystallographic orientation to PIXL. The following describes these individual processes in detail.

#### Forward simulation

For each mineral, we calculated the angles (γ and δ) of the diffraction vectors orthogonal to the diffracting planes, ***g***, for each (hkl) that results in a d-spacing within the range 1 keV < E < 15 keV for a two-theta of 158° (*26*, *67*). Given PIXL’s relative x-ray beam and detector locations, a subset of reflections will be recorded for a given instrument-crystal orientation. For a fixed crystal orientation, we define three angles to describe the relative position of PIXL: α, ϕ, and τ. α and ϕ define polar and elevation angles of the incident x-ray beam relative to the crystallographic *c* axis (*26*), while τ describes the rotation of the two PIXL detectors around the x-ray beam axis. Given PIXL angular resolution of approximately ±4° (*26*), we simulated instrument-crystal orientations with an interval of 4° with the rotation τ reduced to the range 0 < τ < 180 due to detector symmetry.

The relative diffraction peak intensities were calculated using Eq. 1

$$I\left(E\right)=A\left(E\right){|F_{\text{khl}}|}^{2}{\left[\frac{1+{\text{cos}}^{2}\left(2\theta \right)}{{\text{sin}}^{2}\left(\theta \right)\text{cos}\left(\theta \right)}\right]}_{\text{hkl}}$$ (1)

$$F_{\text{hkl}}=\sum_{n}f_{n}e^{2\pi i\left(\mathrm{hx}+\mathrm{ky}+\mathrm{lz}\right)}$$ (2)

$$f_{n}=f_{0}+f\prime \left(E\right)+\text{if}\prime \prime \left(E\right)$$ (3)

where *F*_hkl_ is given by Eq. 2 with the energy-dependent atomic scattering factors given by Eq. 3 and drawn from (*68*). *A*(*E*) is an additional scaling factor equal to the calculated intensity of the incident radiation (*64*) with the Rh L intensities replaced by theoretically determined values (fig. S11) (*69*). Once a reflection is recorded, the structure factor is defined for the specific diffraction angle, θ, with the proportion of any partially detected diffraction intensities determined. The resulting diffracted intensities observed in the two detectors are then included in the forward simulation LUT for each of the three CaSO_4_ minerals: anhydrite (*33*), gypsum (*34*), and bassanite (*35*). Anhydrite was converted to the space group Cmcm for consistency with previous literature (*46*).

#### Identifying diffraction peaks

Diffraction was identified by comparing the two detector outputs for each beam location (*70*, *71*), using diffraction identified by a machine learning algorithm (*26*) as a guide. Once the location of the diffraction was identified, a Gaussian peak was fitted to the difference in counts between the two detectors (detector A − detector B) with a width defined by the detector resolution and an amplitude in counts centered on the peak energy. A positive amplitude denotes a peak in detector A, while a negative amplitude denotes a peak in detector B. For each beam position, the list of identified diffraction peaks was used to create a measured diffraction spectrum with intensities in counts. Photon shot noise, in the form of Poisson noise, was then added to these spectra before the spectra were normalized (Fig. 5, D and E).

#### Mineral identification

The measured diffraction spectra, Ψ(*E*), for each beam location in the CaSO_4_ regions were compared to the simulated diffraction spectra in the LUT for each of the three minerals, Λ(*E*), by calculating the normalized cross-correlation (*r*)

$$r=\frac{1}{n-1}\sum_{E}\frac{\left[\Psi \left(E\right)-\mu_{\Psi }\right]\left[\Lambda \left(E\right)-\mu_{\Lambda }\right]}{\sigma_{\Psi }\sigma_{\Lambda }}$$ (4)

calculated over *n* energy points, *E*, where μ and σ are the mean and SD, respectively. The values for *r* were ranked, and the simulated diffraction spectra with the highest ranked correlation were selected. Because of crystal and instrument symmetries, multiple simulated spectra resulted in the same correlation in many cases.

The highest *r* values for each mineral were compared by applying a Fisher transformation (*72*)

r′=atanh(r) (5)

and comparing the resulting normal distributions

$$z=\frac{\left({r\prime }_{1}-{r\prime }_{2}\right)}{S}$$ (6)

where

$$S=\sqrt{\frac{1}{n_{1}-3}+\frac{1}{n_{2}-3}}$$ (7)

with *n*_1_ and *n*_2_ being the number of observations in *r*_1_ and *r*_2_, respectively. One mineral was selected when the resulting *P* value was ≤ 0.05. A *P* value > 0.05 indicates that a single mineral could not be reliably identified, with these beam locations omitted from further analysis. Furthermore, because of the difficulty phasing bassanite (*13*), beam locations that resulted in bassanite selection were also omitted (18 and 14% of beam locations for BH and UI, respectively).

#### Crystallographic orientation and mapping regional crystalline alignment

The crystal orientation relative to PIXL can be determined from the simulated diffraction spectra through the application of an orientation matrix on the three crystallographic axes where the angles α, ϕ, and τ (*26*) correspond to the Euler angles Φ, φ_2_, and φ_1_ in the Bunge convention (*73*). Regional crystalline alignment is calculated by comparing the *n* determined crystallographic orientations, *A_i_*, for a given PMC with the *m* crystallographic directions, *B_j_*, for the four nearest neighbors using the absolute value of the cosine similarity, ∣SC∣, where SC is defined as

$${\text{SC}}_{i,j}\left(A_{i},B_{j}\right)=\frac{A_{i}\cdot B_{j}}{\left\|A_{i}\right\|\left\|B_{j}\right\|}$$ (8)

The cosine similarity, SC, returns unity for vectors pointing in the same direction, zero for perpendicular vectors, and negative unity for vectors that are pointing in opposite directions. Taking the absolute value of the cosine similarity removes the directionality of the alignment, and the output therefore ranges from zero for perpendicular alignment to one for parallel alignment. The regional crystalline alignment and confidence are then calculated as the average and variance of ∣SC∣, weighted by the inverse of the distance between the given PMC and its three nearest neighbors. Because of the spatial and spectral resolution of PIXL as an XRD instrument (see the Supplementary Materials), this analysis cannot distinguish between a single large crystal and multiple smaller crystals with the same orientation.

### Fracture analysis

We hand-digitized outcrop-scale fracture traces at BH and UI (Fig. 3) as polyline segments and exported the resulting maps as vector files (in SVG format) for orientation analysis with the program FracPaq (*74*). Fractures in the abrasion patch at UI (Fig. 4) were hand-digitized as polygons. The resulting map was exported as binary raster image and skeletonized with ImageJ (*75*). The skeletonized binary raster image was also analyzed with FracPaq. We manually measured the local strike direction of exposed vein segments within the outcrop-scale fracture networks at the points shown in Fig. 3 and used to create the related polar histograms. The horizontal principal stress directions were determined by maximizing the dilation tendency on the entire outcrop-scale fracture network (*42*), using the orientations of the individual polyline segments of the digitized fracture network delivered by FracPaq.

## Supplementary Materials

### This PDF file includes:

Supplementary Text Figs. S1 to S11 Table S1 References
