# Supplementary material for: In situ crystallographic mapping constrains sulfate precipitation and timing in Jezero crater, Mars
Source: Sci Adv. 2025 Apr 16;11(16):eadt3048. doi: 10.1126/sciadv.adt3048 (PMC12002120; doi:10.1126/sciadv.adt3048)
Supplement: Supplementary file 1 — Supplementary Text Figs. S1 to S11 Table S1 References [file sciadv.adt3048_sm.pdf]

Science Advances
AAAS

Supplementary Materials for

In situ crystallographic mapping constrains sulfate precipitation and timing in Jezero crater, Mars

Michael W. M. Jones *et al.*

Corresponding author: Michael W. M. Jones, mw.jones@qut.edu.au

Sci. Adv. **11**, eadt3048 (2025)
DOI: 10.1126/sciadv.adt3048

This PDF file includes:

Supplementary Text
Figs. S1 to S11
Table S1
References

Supplementary Text

Comparison between CheMin and PIXL as XRD instruments

Unlike the *Curiosity* rover, *Perseverance* does not have a dedicated XRD instrument. CheMin, the XRD instrument on *Curiosity*, uses a Co X-ray source (E = 6931eV; λ = 1.79Å) with a 2θ range of 5° to 50° at an angular resolution of <0.35° to obtain transmission X-ray diffraction on grain sizes <150 μm with a nominal detection limit of 3 wt% (*76*). Piezoelectric actuators "shake" the sample to ensure all reflection are recorded so the data can be interpreted analogous to a laboratory powder XRD instrument (*76*).

In contrast, PIXL was designed as an XRF mapping instrument. However, X-ray diffraction peaks are detected in its two energy dispersive detectors and can be separated from the fluorescence signal (*2, 26, 77*). PIXL data comprises of 4096 channels each with an energy width of ~7.88eV and a spectral resolution of ~160 eV FWHM (*25*), giving a peak width of approximately 20 channels. At PIXLs 2θ of 158° (*25, 26*), *overlapping* diffraction peaks can be separated with a 2θ angular resolution of <15°, in terms of a Co X-ray source. However, the energy of a Gaussian peak fitted to *isolated* diffraction peaks can be determined conservatively to an accuracy of 1.5 channels (*78*), giving a 2θ angular resolution of <1° in terms of a Co X-ray source. In practice, this means that single crystals with isolated diffraction peaks can be readily phased with PIXL, given the accurate elemental information, which reduces the number of possible phases significantly. However, as the number of isolated diffraction peaks is reduced due to either the inherent properties of the mineral, a mix of minerals, or multiple smaller crystals at different orientations, the ability of PIXL to make an accurate determination is significantly reduced.

Significance of differences in fracture-wall roughness at Berry Hollow and Uganik Island

The fractures and veins at Wildcat Ridge (abrasion patch Berry Hollow) are generally narrower with straighter margins than those at Hidden Harbor. Since the fracture roughness of rocks generally decreases with decreasing grain size (*79*), this difference could be explained by textural differences in the host rock, which is finer-grained in Berry Hollow compared to Uganik Island (*7*). Alternatively, the degree of lithification at the time of fracture formation could have been different. Therefore, it is possible that the fracture network at Wildcat Ridge either formed at greater burial depth than that at Hidden Harbor or the difference is due to the finer grain size at Wildcat Ridge.

Causes for vertical maximum principal stress, the preferred orientation of veins, and the triaxial stress state

A vertically oriented maximum principal stress, as derived for the fracture networks at Berry Hollow and Uganik Island, is expected in a laterally constrained layer of rock under gravitational loading due to Poisson's effect (e.g., (*43*)) and does not require tectonic forces. Interestingly, the fracture traces, and much more so the vein traces, at both outcrops show a preferred orientation in the horizontal plane (**Fig. 3**). This preferential orientation of vein segments could be due to observational bias. The most frequently observed vein orientations are subparallel to the local topographic downhill direction (**Fig. 3**). Therefore, the mapped vein segments could have been exposed preferentially due to wind moving along the slope, biasing the documented orientation distribution. However, the veins could have also formed in a true triaxial state of stress ($\sigma 1 > \sigma 2 > \sigma 3$), even in the absence of tectonic forces. A deviation from perfect isotropy of fracture trace orientation is observed in experimental desiccation cracks formed in laterally unconstrained layers and can be easily produced by lateral changes in layer thickness, material properties (density, tensile strength, friction coefficient, pore-fluid pressure, moisture, etc.), basal

friction with the underlying sedimentary beds, and geometry of the basal (or overlying) bed interface (*80*).

Further discussion of vein textures in the Berry Hollow wing crack

The geometry of the vein studied in detail at Berry Hollow resembles a wing crack (**Figs. 2, 5**), which implies that the central vein segment experienced both an opening and shear displacement. The crystal texture with its notable crystallographic preferred orientation (CPO) supports this interpretation. On Earth, syntaxial veins with elongate-blocky fabric and stretching veins have been observed to display a CPO (*47*). These vein fabrics are attributed to crack-seal veins formed under deviatoric stress (*47*). In addition, terrestrial gypsum and anhydrite commonly form mineral fibers in veins in antitaxial veins (*47, 81*), which can also exhibit a CPO. However, this CPO is weaker than that documented at Berry Hollow. In fibrous gypsum, also known as satin spar, the a-axes are aligned but with random rotations of the b- and c-axes around the a-axis (*82*). Fibrous anhydrite displays similar behavior with an alignment of the c-axes alone (*81*). Due to the lack of this rotational symmetry, we interpret the crystal texture of the vein at Berry Hollow as that of a crack-seal vein rather than an antitaxial vein. Interestingly, the [010] planes of gypsum, and those of anhydrite, strike approximately parallel to the strike expected from shear fractures formed under the stress state derived from the wing-crack geometry (**Fig. 5**). A similar CPO has been observed in deformation experiments on natural rehydrating anhydrite that is compacted under constant differential stress (*46*). Given the parabolic shape of the brittle yield envelope for rocks in the tensile domain, this also means that this vein formed at greater differential stress (e.g., Figure 3 in Ref. (*47*)), and thus greater burial depth compared to the vertical extension fractures observed elsewhere. Hence, it is also possible that some of the larger fractures mapped at the surface of Wildcat Ridge are also mixed-mode fractures which should have a dip angle < 90°.

Estimation of burial depth

The grain sizes at Berry Hollow and Uganik Island correspond to very fine to fine grained sand, and very fine to medium grained sand respectively (*7, 83*). The tensile strength (T_s) for fine grained sandstones varies, depending on the degree of cementation, and ranges from 75 kPa to 750 kPa for weakly cemented sandstones (*52*). For mixed-mode fractures such as the wing crack at the Berry Hollow abrasion, $\Delta\sigma > 4\ T_s$ (*41*). Therefore, for weakly cemented sandstones, as described above (*52*), we obtain minimum burial depths between 80 and 800 m with a Poisson's ratio for fine grained sandstone of ~0.29 (*84*) and a density of 1.68 gcm^{-3} (*85*) (**fig. S10**). Using geomorphological evidence and crater counting, it has been estimated that ~ 70 m of material has been removed from above the crater floor (*55*). The outcrops studied here are within ~10 - 20 vertical meters of the crater floor contact (*6*). Our quantitative estimates of minimum burial depth are thus in close agreement with the lower bound estimated by these analyses.

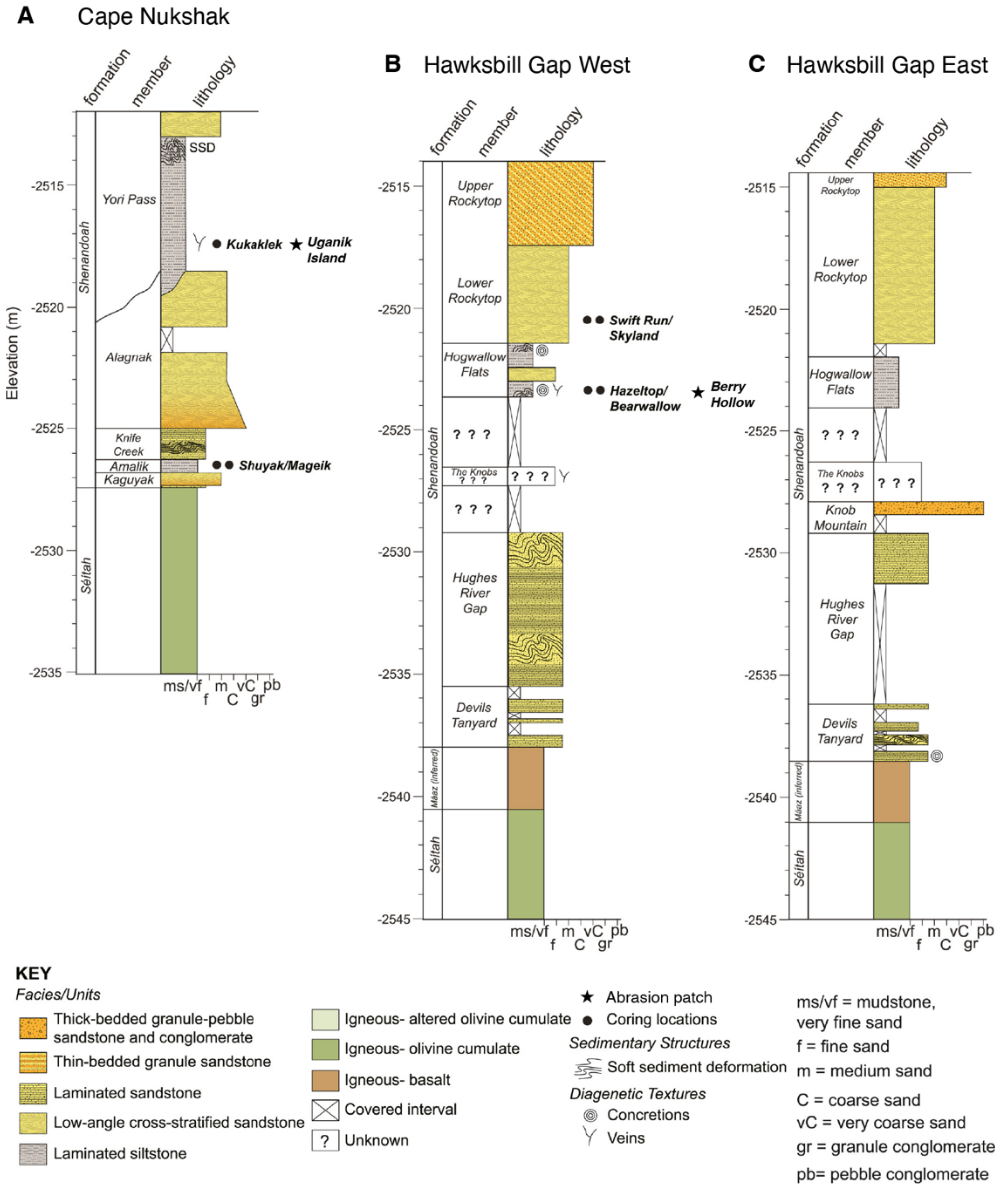

Fig. S1. Stratigraphy of the Shenandoah formation. Stratigraphic columns for the Shenandoah formation reproduced with permission from Stack *et al*., (2024) (*6*). The Uganik Island and Berry Hollow abrasions are denoted by stars in **A** and **B** respectively.

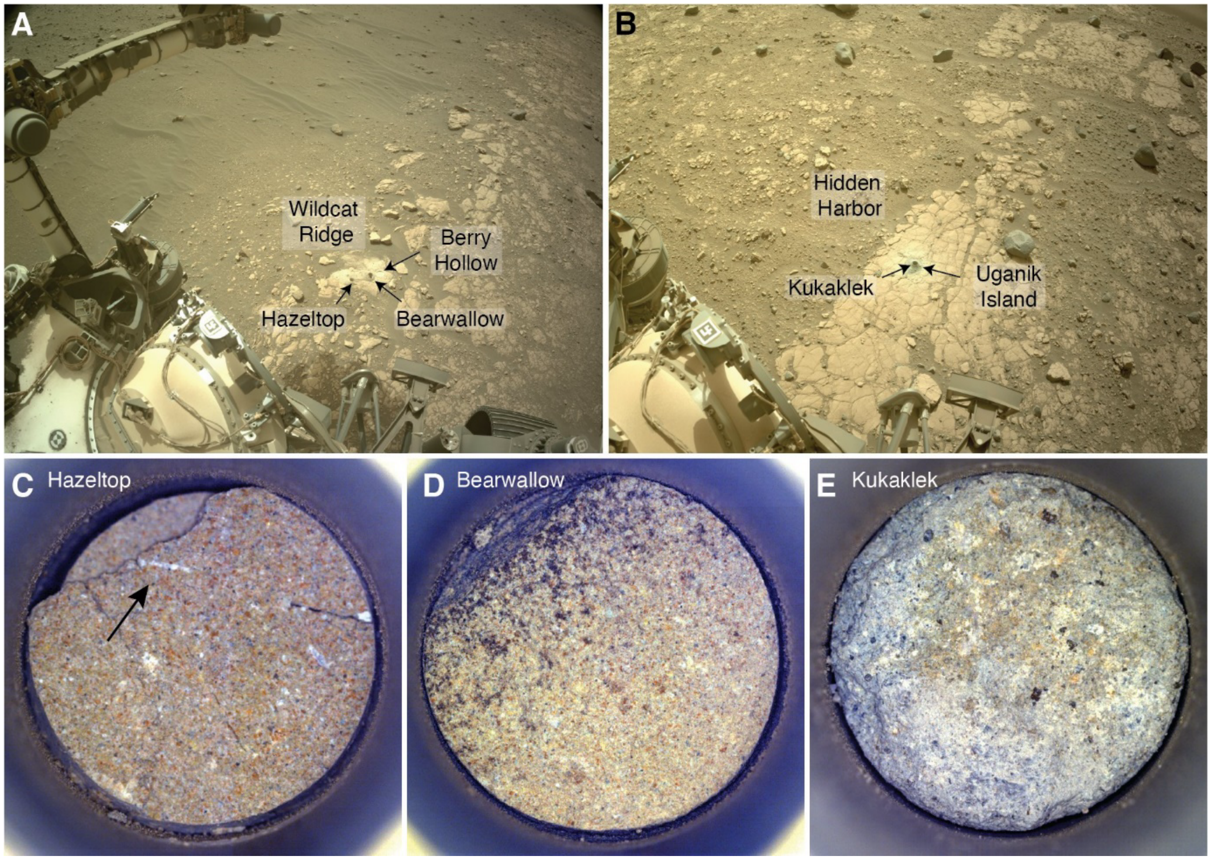

Fig. S2. Location of the core samples relative to the abrasion and outcrop. Location of the three cores for Mars Sample Return (*5*) at Wildcat Ridge (Hogwallow Flats member) (**A**) and Hidden Harbor (Yori Pass member) (**B**). Cachecam (*86*) (**C-E**) images of the Hazeltop (sealed on sol 509), Bearwallow (sealed on sol 516), and Kukaklek (sealed on sol 631) cores in the bit. The diameter of each rock core is 13 mm. A clear sulfate vein is visible in the Cachecam image of the Hazeltop core sample (black arrow, C), similar to that observed at Berry Hollow (**Figs. 1, 2, 5**; **fig. S3**).

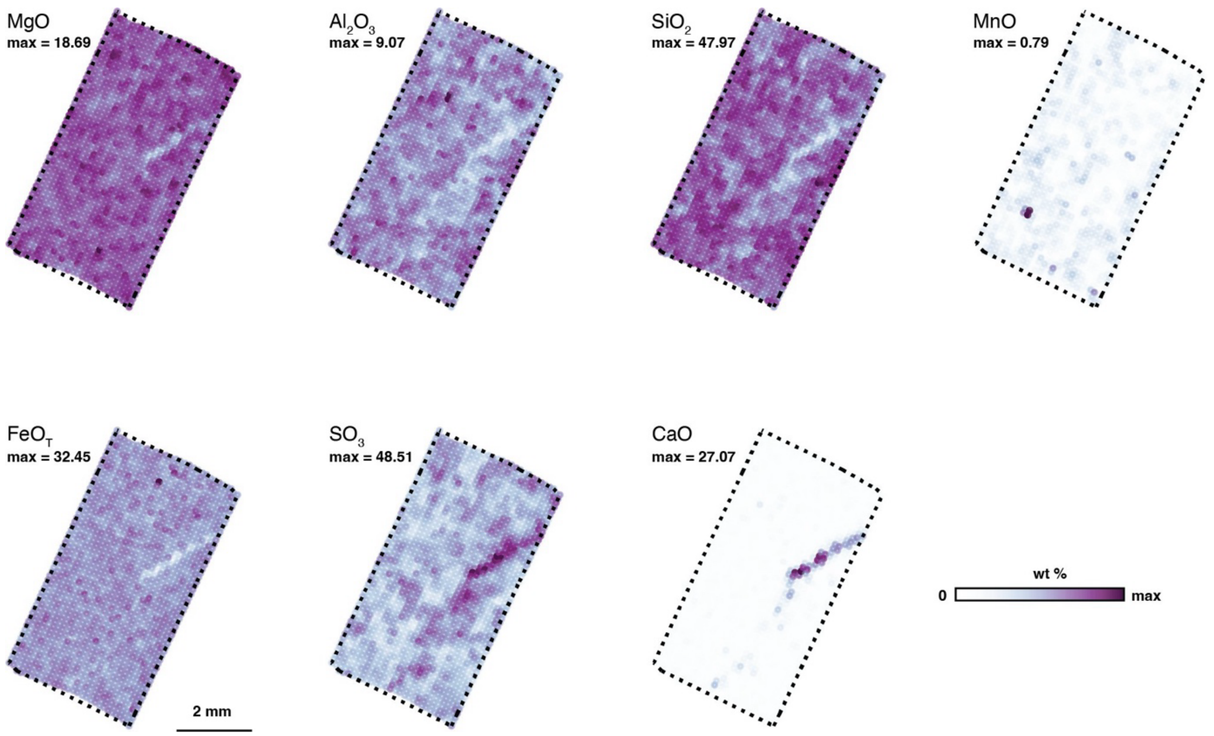

Fig. S3. Elemental Maps for BH2. Concentration maximum is listed for each panel. All panels have the same zero point, with the colormap referring to all panels. The location of each scan is shown in **Fig. 1D** and **Fig. 2A**.

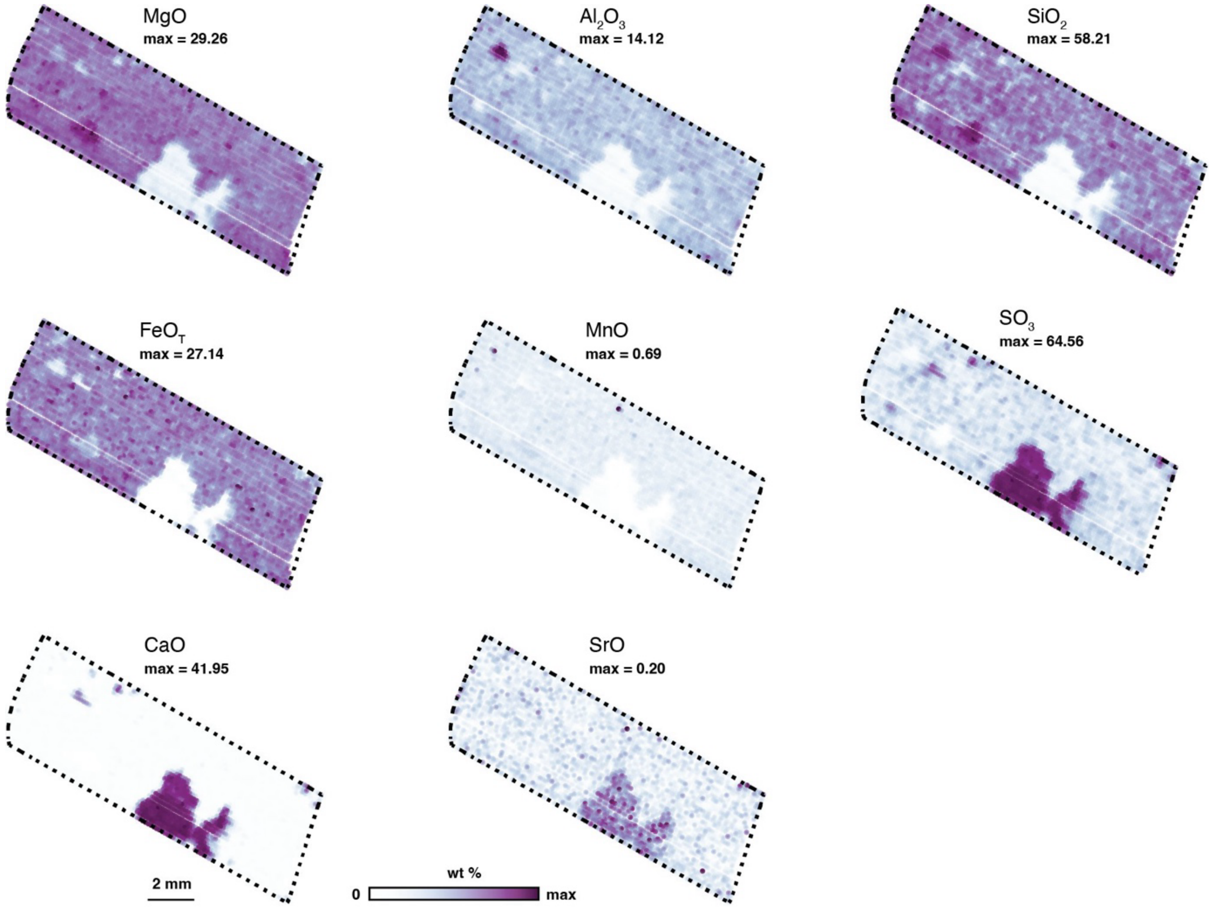

Fig. S4. Elemental Maps for UI1. Concentration maximum is listed for each panel. All panels have the same zero point, with the colormap referring to all panels. The location of each scan is shown in **Fig. 1F** and **Fig. 2B**.

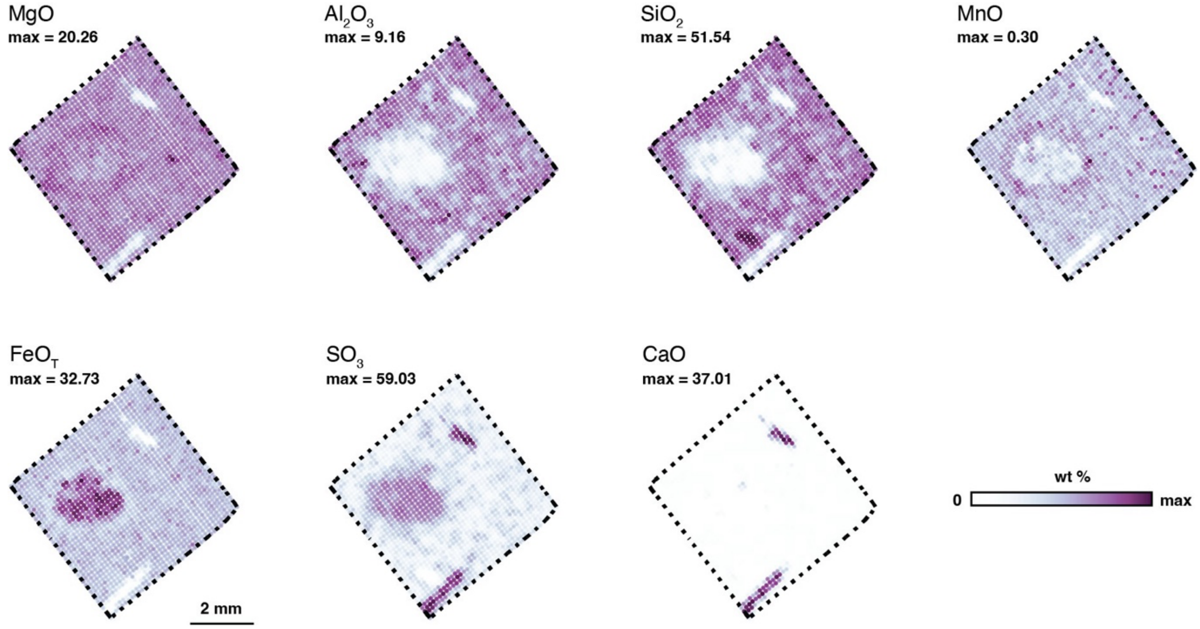

Fig. S5. Elemental Maps for UI2. Concentration maximum is listed for each panel. All panels have the same zero point, with the colormap referring to all panels. The location of each scan is shown in **Fig. 1F**.

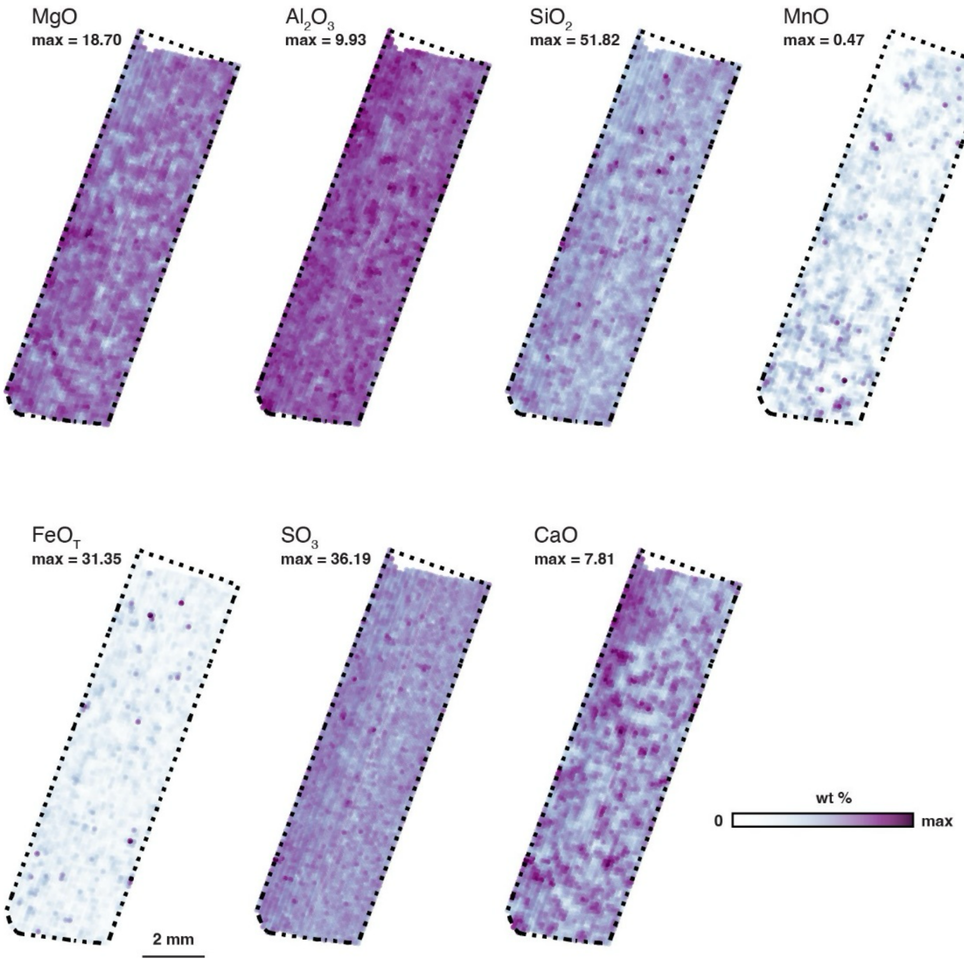

Fig. S6. Elemental Maps for BH1. Concentration maximum is listed for each panel. All panels have the same zero point, with the colormap referring to all panels. The location of each scan is shown in **Fig. 1D**.

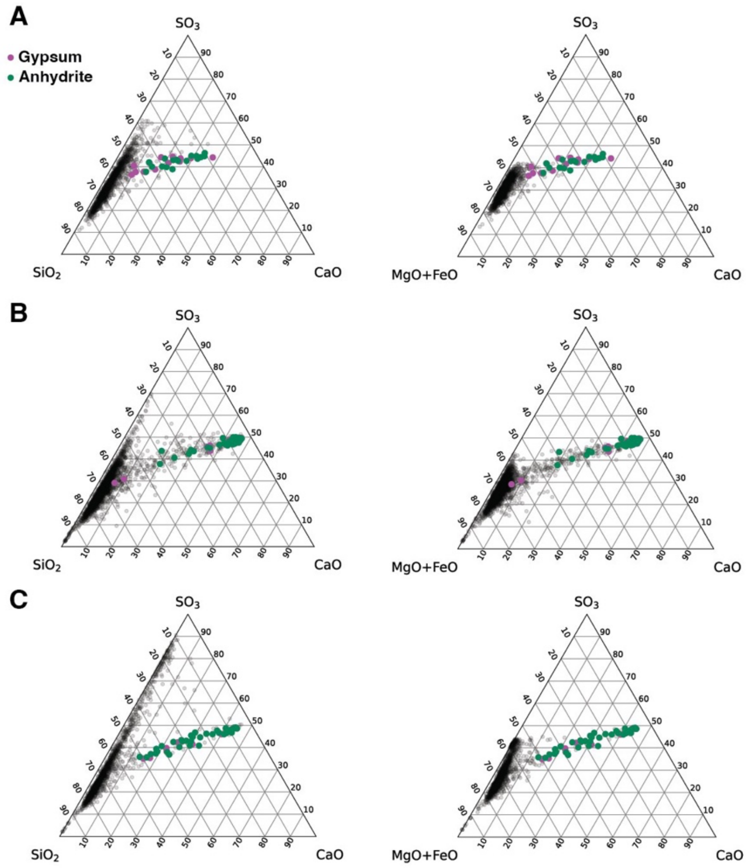

Fig. S7. Ternary diagrams using molar abundances. Ternary diagrams for BH2 (**A**), UI1 (**B**), and UI2 (**C**). In each panel, all PMCs are in grey, with gypsum and anhydrite identified in **Fig. 2** (BH2 and UI1) and **fig. S8** (UI2) overlaid in purple and green respectively.

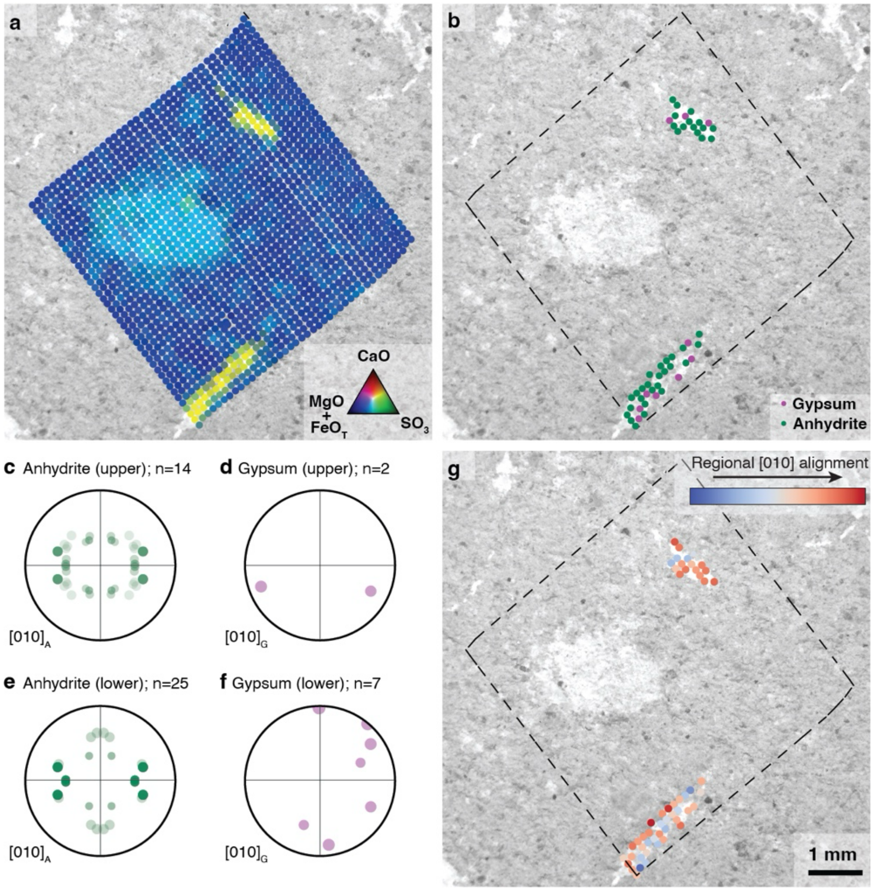

Fig. S8. Elemental, mineral, and crystallographic orientation mapping. PIXL results for UI2 shown overlaid on a SHERLOC ACI image. The scan location is shown in **Fig. 1F**. Three color RGB image of CaO (max 6.6 mmolg^{-1}), SO_3 (max 7.3 mmolg^{-1}), and FeO_T + MgO (max 7.7 mmolg^{-1}) (**A**). $CaSO_4$ minerals appear yellow according to the color mixing triangle in (**A**) confirms that $CaSO_4$ minerals are present in the light toned regions. Mineral identification map (**B**) shows that anhydrite is the dominant phase. The mineral proportions are 70% anhydrite, 18% gypsum, 12% bassanite. As discussed elsewhere, locations identified as bassanite were discarded from further analysis. Random [010] pole figures for both upper (**C, D**) and lower (**E, F**) features over the region of interest indicates no CPO in either case. Mapping the regional alignment of the [010] plane for both anhydrite and gypsum (**G**) reveals regions of high crystallographic alignment throughout the areas, suggesting a crystalline blocky texture. The scale bar in (**G**) also applies to (**A**) and (**B**).

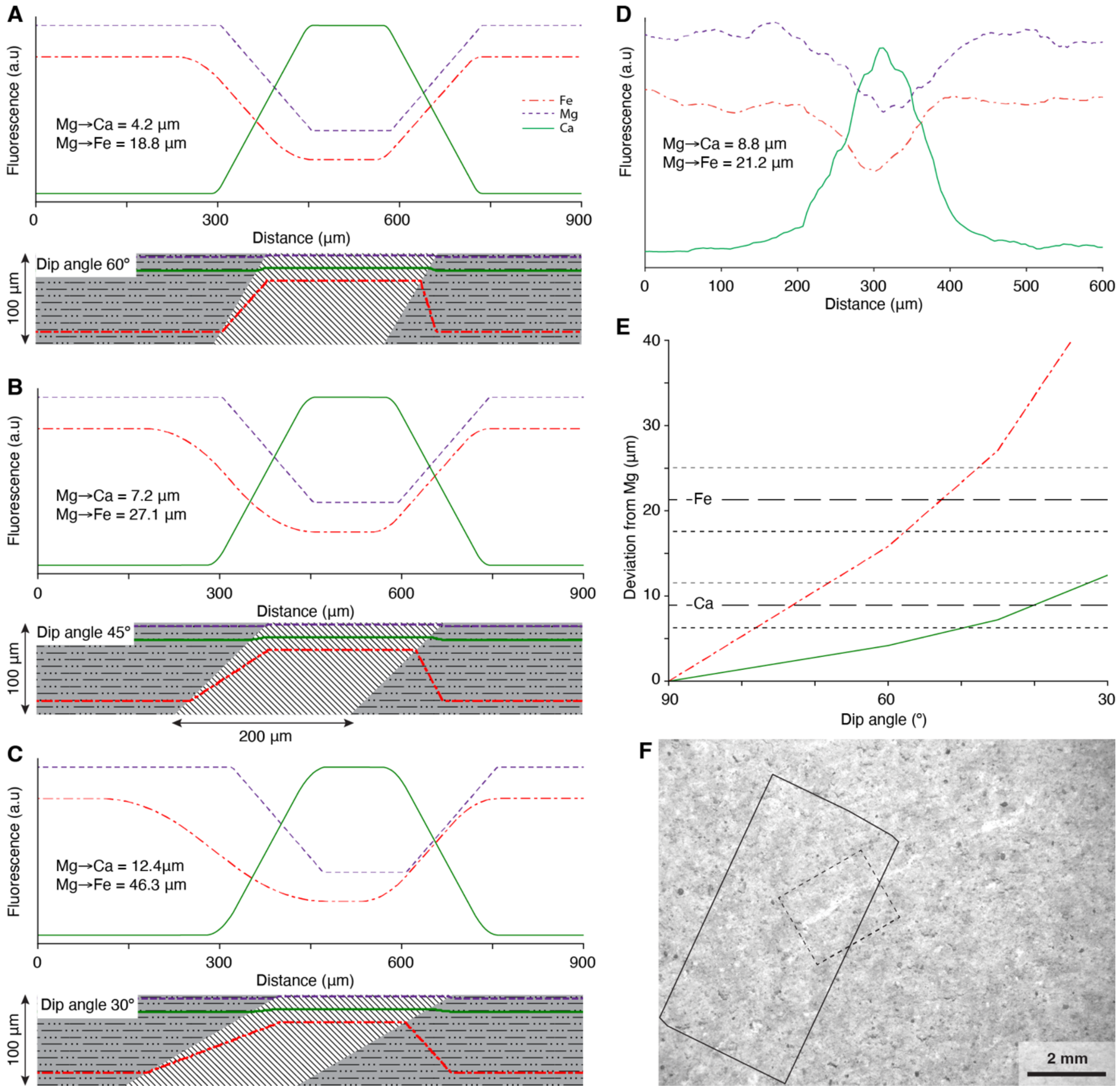

Fig. S9. Dip angle analysis. Simulations of the effect of vein dip angles on the fluorescent signal for a calcium sulfate (modelled as $CaSO_4$, $\rho = 2.32$ gcm^{-3}) vein with a surface exposure of 200 µm in a Fe-Mg rich silica host rock (modelled as SiO_2 with Gale crater bedrock density, $\rho = 1.68$ gcm^{-3} (*85*)). Absorption coefficients from Elam *et al*., 2002 (*87*). The profile of the expected fluorescence yield is dependent on the maximum escape depth of the fluorescent photons (lines plotted on schematics) and the dip angle of the vein. With a shallow escape depth, Mg accurately measures the vein exposed at the surface. As the escape depth increases the fluorescent signal maps more of the subsurface vein, therefore shifting towards the direction of the vein dip. For the case of a host rock density of 1.68 gcm^{-3} and a vein dip of 30° to the surface (**A**), Ca and Fe are shifted 9.7 and 33.9 µm relative to Mg, respectively. For the case of a vein dip of 45° and 60° to the surface (**B, C**), Ca and Fe are shifted 3.2, 5.6 and 11.4, 19.7 µm respectively. For a vertical vein with a dip of 90° to the surface, all there is no shift between the various elemental profiles. Analysis of the vein at BH (**D**) shows that Ca and Fe are shifted 8.8 and 21.2 µm relative to Mg, respectively. Plots of the deviation from Mg as a function of dip angle for Fe and Ca with a host rock density of 1.68 gcm^{-3} (**E**). The dashed horizontal lines indicate the fitted deviation from Mg for Fe and Ca (**D**), with the dotted lines indicating the 95% confidence bounds of the fitted peak position. Comparing fitted profiles for the vein segment at BH2 (**D**) and the simulated data (**E**),

we find a dip angle relative to the surface of ~ 50°. (**F**) shows the location of the profiles in (**D**) (dashed box) and the extent of the scan area (solid box).

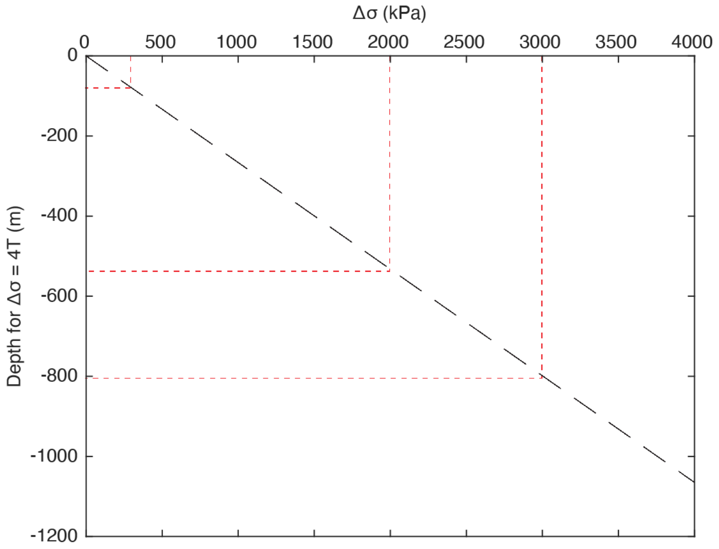

Fig. S10. Horizontal stress and burial depth relationship. Relationship between the difference of the vertical and horizontal stress components ($\Delta\sigma$) and the burial depth using the following parameters: rock density ρ = **1.68 gcm^{-3} (*85*)**, gravitational acceleration g = 3.73 ms^{-2}, Poisson's ratio n = 0.29 (for fine grained sediment (*84*)). Letting h denote burial depth, $\Delta\sigma$ is calculated as (*41*): $\Delta\sigma = \rho g h \left(1 - \frac{1}{(n^{-1}-1)}\right)$. For tensile strengths (T) of 75 kPa, 500 kPa and 750 kPa, (Δσ = 4T = 300 kPa, 2 MPa and 3 MPa) (*52*) we obtain burial depths of 80 m, ~530m and 800 m, respectively (dashed red lines).

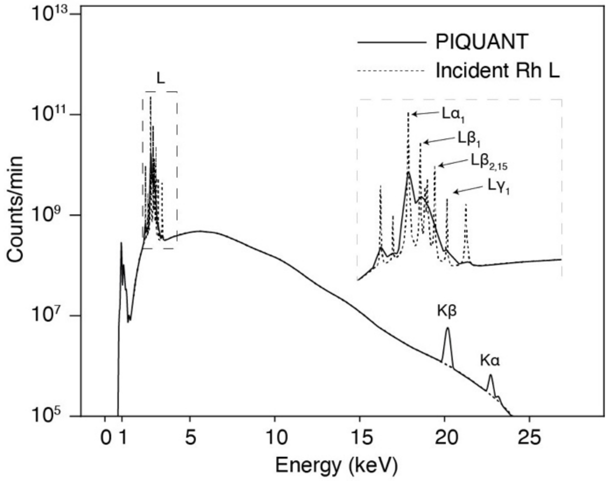

Fig. S11. Rh L emission line spectrum modeling. Incident intensity spectrum is most intense around the Rh L emission lines, with a highly non uniform intensity. This leads to relative

increased intensities of some diffraction peaks, notably anhydrite (022) excited by the $L\alpha_1$ peak at 2696.8 eV (inset).

Table S1: Image file names and sources. All images are available in the NASA Planetary Data System https://pds.nasa.gov/ under the following dois. PIXL data: doi:10.17189/1522645. Image data from the following cameras was used (see Table S1 for specific image names): HiRISE images: doi:10.17189/1520303, Navcam (image prefix NLF) and Cachecam (image prefix CCF): doi:10.17189/1522847, SHERLOC Imaging (image prefix SI1, SC3, SIF): doi:10.17189/1522846, Mastcam-Z (image prefix ZLF, ZRF): doi:10.17189/1522843.

**Fig. 1**	
1A	HiRISE Color Basemap v5p1 NASA/JPL/University of Arizona
1B	HiRISE Color Basemap v5p1 NASA/JPL/University of Arizona
1C	NLF_0612_0721280802_722FDR_N0301172NCAM00709_0A1095J03 (*86*)
1D	SI1_0612_0721292646_167FDR_N0301172SRLC00033_000095J02 (*88*)
1E	NLF_0504_0711684404_926FDR_N0261222NCAM00709_0A0095J01 (*86*)
1F	SIF_0504_0711696434_832FDR_N0261222SRLC02504_0000LMJ01 (*88*)
**Fig. 2**	
2A	SC3_0614_0721480198_003FDR_N0301172SRLC10600_0000LMJ01 (*88*)
2B	SC3_0513_0712520578_316FDR_N0261222SRLC16015_0000LMJ01 (*88*)
**Fig. 3**	
3A	ZRF_0612_0721272628_348RAS_N0301172ZCAM03480_1100LMJ01 (*89*)
3B	ZLF_0502_0711509150_010FDR_N0261222ZCAM07101_1100LMJ01 (*89*)<br>This image is an oblique view of the outcrop. To reduce angular errors, and to align it with the other representations of the outcrop, it has been transformed with the following transformation matrix:<br><br>[2.43726, 0.19372, 0.00017]<br>[0.29979, 3.35840, 0.00033]<br>[-1204.20588, -786.28054, 1.0]
**Fig. 4**	
4A	SI1_0612_0721292646_167FDR_N0301172SRLC00033_000095J02 (*88*)
4D	SC3_0614_0721480198_003FDR_N0301172SRLC10600_0000LMJ01 (*88*)
**Fig. 5**	SC3_0513_0712520578_316FDR_N0261222SRLC16015_0000LMJ01 (*88*)
**Fig. S3**	
S3A	NLF_0527_0713726823_925FDR_N0261222NCAM00712_0A0095J01 (*86*)
S3B	NLF_0626_0722520924_850FDR_N0301172NCAM00709_0A0095J01 (*86*)
S3C	CCFC0509_0712155363_000FDR_N0261222CACH00105_0A00LLJ01-s3 (*86*)
S3D	CCFC0516_0712775180_000FDR_N0261222CACH00105_0A00LLJ01-s3 (*86*)
S3E	CCF_0626_0722537762_729FDR_N0301172CACH00228_0M00LLJ01-s3 (*86*)
**Fig. S9**	SC3_0617_0721745135_414FDR_N0301172SRLC10600_0000LMJ01 (*88*)
**Fig. S10**	SC3_0513_0712520578_316FDR_N0261222SRLC16015_0000LMJ01 (*88*)

REFERENCES AND NOTES

1. K. A. Farley, K. M. Stack, D. L. Shuster, B. H. N. Horgan, J. A. Hurowitz, J. D. Tarnas, J. I. Simon, V. Z. Sun, E. L. Scheller, K. R. Moore, S. M. Mc Lennan, P. M. Vasconcelos, R. C. Wiens, A. H. Treiman, L. E. Mayhew, O. Beyssac, T. V. Kizovski, N. J. Tosca, K. H. Williford, L. S. Crumpler, L. W. Beegle, J. F. Bell III, B. L. Ehlmann, Y. Liu, J. N. Maki, M. E. Schmidt, A. C. Allwood, H. E. F. Amundsen, R. Bhartia, T. Bosak, A. J. Brown, B. C. Clark, A. Cousin, O. Forni, T. S. J. Gabriel, Y. Goreva, S. Gupta, S.-E. Hamran, C. D. K. Herd, K. Hickman-Lewis, J. R. Johnson, L. C. Kah, P. B. Kelemen, K. B. Kinch, L. Mandon, N. Mangold, C. Quantin-Nataf, M. S. Rice, P. S. Russell, S. Sharma, S. Siljeström, A. Steele, R. Sullivan, M. Wadhwa, B. P. Weiss, A. J. Williams, B. V. Wogsland, P. A. Willis, T. A. Acosta-Maeda, P. Beck, K. Benzerara, S. Bernard, A. S. Burton, E. L. Cardarelli, B. Chide, E. Clavé, E. A. Cloutis, B. A. Cohen, A. D. Czaja, V. Debaille, E. Dehouck, A. G. Fairén, D T Flannery, S Z Fleron, T. Fouchet, J. Frydenvang, B J Garczynski, E F Gibbons, E M Hausrath, A G Hayes, J. Henneke, J L Jørgensen, E. M. Kelly, J. Lasue, S. Le Mouélic, J. M. Madariaga, S. Maurice, M. Merusi, P.-Y. Meslin, S. M. Milkovich, C. C. Million, R. C. Moeller, J. I. Núñez, A. M. Ollila, G. Paar, D. A. Paige, D. A. K. Pedersen, P. Pilleri, C. Pilorget, P. C. Pinet, J. W. Rice Jr, C. Royer, V. Sautter, M. Schulte, M. A. Sephton, S. K. Sharma, S. F. Sholes, N. Spanovich, M. St. Clair, C. D. Tate, K. Uckert, S. J. Van Bommel, A. G. Yanchilina, M.-P. Zorzano, Aqueously altered igneous rocks sampled on the floor of Jezero crater, Mars. *Science* **377**, eabo2196 (2022).

2. Y. Liu, M. M. Tice, M. E. Schmidt, A. H. Treiman, T. V. Kizovski, J. A. Hurowitz, A. C. Allwood, J. Henneke, D. A. K. Pedersen, S. J. VanBommel, M. W. M. Jones, A. L. Knight, B. J. Orenstein, B. C. Clark, W. T. Elam, C. M. Heirwegh, T. Barber, L. W. Beegle, K. Benzerara, S. Bernard, O. Beyssac, T. Bosak, A. J. Brown, E. L. Cardarelli, D. C. Catling, J. R. Christian, E. A. Cloutis, B. A. Cohen, S. Davidoff, A. G. Fairén, K. A. Farley, D. T. Flannery, A. Galvin, J. P. Grotzinger, S. Gupta, J. Hall, C. D. K. Herd, K. Hickman-Lewis, R. P. Hodyss, B. H. N. Horgan, J. R. Johnson, J. L. Jørgensen, L. C. Kah, J. N. Maki, L. Mandon, N. Mangold, F. M. McCubbin, S. M. McLennan, K. Moore, M. Nachon, P. Nemere, L. D. Nothdurft, J. I. Núñez, L. O'Neil, C. M. Quantin-Nataf, V. Sautter, D. L. Shuster, K. L. Siebach, J. I. Simon, K. P. Sinclair, K. M. Stack, A. Steele, J. D. Tarnas, N. J. Tosca, K. Uckert, A. Udry, L. A. Wade, B. P. Weiss, R. C.

Wiens, K. H. Williford, M.-P. Zorzano, An olivine cumulate outcrop on the floor of Jezero crater, Mars. *Science* **377**, 1513–1519 (2022).

3. N. Mangold, S. Gupta, O. Gasnault, G. Dromart, J. D. Tarnas, S. F. Sholes, B. Horgan, C. Quantin-Nataf, A. J. Brown, S. Le Mouélic, R. A. Yingst, J. F. Bell, O. Beyssac, T. Bosak, F. Calef, B. L. Ehlmann, K. A. Farley, J. P. Grotzinger, K. Hickman-Lewis, S. Holm-Alwmark, L. C. Kah, J. Martinez-Frias, S. M. McLennan, S. Maurice, J. I. Nuñez, A. M. Ollila, P. Pilleri, J. W. Rice, M. Rice, J. I. Simon, D. L. Shuster, K. M. Stack, V. Z. Sun, A. H. Treiman, B. P. Weiss, R. C. Wiens, A. J. Williams, N. R. Williams, K. H. Williford, Perseverance rover reveals an ancient delta-lake system and flood deposits at Jezero crater, Mars. *Science* **374**, 711–717 (2021).

4. K. A. Farley, K. H. Williford, K. M. Stack, R. Bhartia, A. Chen, M. de la Torre, K. Hand, Y. Goreva, C. D. K. Herd, R. Hueso, Y. Liu, J. N. Maki, G. Martinez, R. C. Moeller, A. Nelessen, C. E. Newman, D. Nunes, A. Ponce, N. Spanovich, P. A. Willis, L. W. Beegle, J. F. Bell, A. J. Brown, S.-E. Hamran, J. A. Hurowitz, S. Maurice, D. A. Paige, J. A. Rodriguez-Manfredi, M. Schulte, R. C. Wiens, Mars 2020 mission overview. *Space Sci. Rev.* **216**, 142 (2020).

5. M. A. Meyer, G. Kminek, D. W. Beaty, B. L. Carrier, T. Haltigin, L. E. Hays, C. B. Agree, H. Busemann, B. Cavalazzi, C. S. Cockell, V. Debaille, D. P. Glavin, M. M. Grady, E. Hauber, A. Hutzler, B. Marty, F. M. McCubbin, L. M. Pratt, A. B. Regberg, A. L. Smith, C. L. Smith, R. E. Summons, T. D. Swindle, K. T. Tait, N. J. Tosca, A. Udry, T. Usui, M. A. Velbel, M. Wadhwa, F. Westall, M.-P. Zorzano, Final Report of the Mars Sample Return Science Planning Group 2 (MSPG2). *Astrobiology* **22**, S5–S26 (2022).

6. K. M. Stack, L. R. W. Ives, S. Gupta, M. P. Lamb, M. Tebolt, G. Caravaca, J. P. Grotzinger, P. Russell, D. L. Shuster, A. J. Williams, H. Amundsen, S. Alwmark, A. M. Annex, R. Barnes, J. Bell III, O. Beyssac, T. Bosak, L. S. Crumpler, E. Dehouck, S. J. Gwizd, K. Hickman-Lewis, B. H. N. Horgan, J. Hurowitz, H. Kalucha, O. Kanine, C. Lesh, J. Maki, N. Mangold, N. Randazzo, C. Seeger, R. M. E. Williams, A. Brown, E. Cardarelli, H. Dypvik, D. Flannery, J. Frydenvang, S.-E. Hamran, J. I. Núñez, D. Paige, J. I. Simon, M. Tice, C. Tate, R. C. Wiens, Sedimentology

and stratigraphy of the Shenandoah Formation, Western Fan, Jezero Crater, Mars. *J. Geophys. Res. Planets* **129**, e2023JE008187 (2024).

7. K. C. Benison, K. K. Gill, S. Sharma, S. Siljeström, M. Zawaski, T. Bosak, A. Broz, B. C. Clark, E. Cloutis, A. D. Czaja, D. Flannery, T. Fornaro, F. Gómez, K. Hand, C. D. K. Herd, J. R. Johnson, J. M. Madariaga, M. B. Madsen, J. Martinez-Frías, M. Nachon, J. I. Núñez, D. A. K. Pedersen, N. Randazzo, D. L. Shuster, J. Simon, A. Steele, C. Tate, A. Treiman, K. Uckert, A. J. Williams, A. Yanchilina, Depositional and diagenetic sulfates of Hogwallow Flats and Yori Pass, Jezero Crater: Evaluating preservation potential of environmental indicators and possible biosignatures from past martian surface waters and groundwaters. *J. Geophys. Res. Planets* **129**, e2023JE008155 (2024).

8. A. P. Broz, B. Horgan, H. Kalucha, J. R. Johnson, C. Royer, E. Dehouck, L. Mandon, E. L. Cardarelli, B. Garczynski, J. H. Haber, K. C. Benison, E. Ives, K. M. Stack, N. Mangold, T. Bosak, J. I. Simon, P. Gasda, E. Clave, B. S. Kathir, M. Zawaski, R. Barnes, S. Siljeström, N. Randazzo, J. M. Madariaga, K. Farley, J. Maki, L. Kah, W. Rapin, L. L. Kivrak, A. J. Williams, E. Hausrath, J. I. Núñez, F. Gómez, A. Steele, T. Fouchet, J. F. Bell, R. C. Wiens, Diagenetic history and biosignature preservation potential of fine-grained rocks at Hogwallow Flats, Jezero Crater, Mars. *J. Geophys. Res. Planets* **129**, e2024JE008520 (2024).

9. H. Wänke, J. Bruckner, G. Dreibus, R. Rieder, I. Ryabchikov, Chemical composition of rocks and soils at the Pathfinder site. *Space Sci. Rev.* **96**, 317–330 (2001).

10. A. S. Yen, R. Gellert, C. Schroder, R. V. Morris, J. F. Bell, A. T. Knudson, B. C. Clark, D. W. Ming, J. A. Crisp, R. E. Arvidson, D. Blaney, J. Bruckner, P. R. Christensen, D. J. DesMarais, P. A. de Souza, T. E. Economou, A. Ghosh, B. C. Hahn, K. E. Herkenhoff, L. A. Haskin, J. A. Hurowitz, B. L. Joliff, J. R. Johnson, G. Klingelhofer, M. B. Madsen, S. M. McLennan, H. Y. McSween, L. Richter, R. Rieder, D. Rodionov, L. Soderblom, S. W. Squyres, N. J. Tosca, A. Wang, M. Wyatt, J. Zipfel, An integrated view of the chemistry and mineralogy of martian soils. *Nature* **436**, 49–54 (2005).

11. J.-P. Bibring, Y. Langevin, J. F. Mustard, F. Poulet, R. Arvidson, A. Gendrin, B. Gondet, N. Mangold, P. Pinet, F. Forget, M. Berthé, J.-P. Bibring, A. Gendrin, C. Gomez, B. Gondet, D.

Jouglet, F. Poulet, A. Soufflot, M. Vincendon, M. Combes, P. Drossart, T. Encrenaz, T. Fouchet, R. Merchiorri, G. Belluci, F. Altieri, V. Formisano, F. Capaccioni, P. Cerroni, A. Coradini, S. Fonti, O. Korablev, V. Kottsov, N. Ignatiev, V. Moroz, D. Titov, L. Zasova, D. Loiseau, N. Mangold, P. Pinet, S. Douté, B. Schmitt, C. Sotin, E. Hauber, H. Hoffmann, R. Jaumann, U. Keller, R. Arvidson, J. F. Mustard, T. Duxbury, F. Forget, G. Neukum, Global mineralogical and aqueous mars history derived from OMEGA/Mars express data. *Science* **312**, 400–404 (2006).

12. S. L. Murchie, J. F. Mustard, B. L. Ehlmann, R. E. Milliken, J. L. Bishop, N. K. McKeown, E. Z. N. Dobrea, F. P. Seelos, D. L. Buczkowski, S. M. Wiseman, R. E. Arvidson, J. J. Wray, G. Swayze, R. N. Clark, D. J. D. Marais, A. S. McEwen, J. P. Bibring, A synthesis of Martian aqueous mineralogy after 1 Mars year of observations from the Mars Reconnaissance Orbiter. *J. Geophys. Res.* **114**, E00D06 (2009).

13. D. T. Vaniman, G. M. Martínez, E. B. Rampe, T. F. Bristow, D. F. Blake, A. S. Yen, D. W. Ming, W. Rapin, P.-Y. Meslin, J. M. Morookian, R. T. Downs, S. J. Chipera, R. V. Morris, S. M. Morrison, A. H. Treiman, C. N. Achilles, K. Robertson, J. P. Grotzinger, R. M. Hazen, R. C. Wiens, D. Y. Sumner, Gypsum, bassanite, and anhydrite at Gale crater, Mars. *Am. Mineral.* **103**, 1011–1020 (2018).

14. R. E. Arvidson, S. W. Squyres, J. F. Bell, J. G. Catalano, B. C. Clark, L. S. Crumpler, P. A. de Souza, A. G. Fairen, W. H. Farrand, V. K. Fox, R. Gellert, A. Ghosh, M. P. Golombek, J. P. Grotzinger, E. A. Guinness, K. E. Herkenhoff, B. L. Jolliff, A. H. Knoll, R. Li, S. M. McLennan, D. W. Ming, D. W. Mittlefehldt, J. M. Moore, R. V. Morris, S. L. Murchie, T. J. Parker, G. Paulsen, J. W. Rice, S. W. Ruff, M. D. Smith, M. J. Wolff, Ancient aqueous environments at Endeavour Crater, Mars. *Science* **343**, 1248097 (2014).

15. J. P. Grotzinger, D. Y. Sumner, L. C. Kah, K. Stack, S. Gupta, L. Edgar, D. Rubin, K. Lewis, J. Schieber, N. Mangold, R. Milliken, P. G. Conrad, D. Des Marais, J. Farmer, K. Siebach, F. Calef III, J. Hurowitz, S. M. Mc Lennan, D. Ming, D. Vaniman, J. Crisp, A. Vasavada, K. S. Edgett, M. Malin, D. Blake, R. Gellert, P. Mahaffy, R. C. Wiens, S. Maurice, J. A. Grant, S. Wilson, R. C. Anderson, L. Beegle, R. Arvidson, B. Hallet, R. S. Sletten, M. Rice, J. Bell III, J. Griffes, B. Ehlmann, R. B. Anderson, T. F. Bristow, W. E. Dietrich, G. Dromart, J. Eigenbrode, A.

Fraeman, C. Hardgrove, K. Herkenhoff, L. Jandura, G. Kocurek, S. Lee, L A Leshin, R. Leveille, D. Limonadi, J. Maki, S Mc Closkey, M. Meyer, M. Minitti, H. Newsom, D. Oehler, A. Okon, M. Palucis, T. Parker, S. Rowland, M. Schmidt, S. Squyres, A. Steele, E. Stolper, R. Summons, A. Treiman, R. Williams, A. Yingst, MSL Science Team, A habitable fluvio-lacustrine environment at Yellowknife Bay, Gale Crater, Mars. *Science* **343**, 1242777 (2014).

16. J. P. Grotzinger, J. A. Crisp, A. R. Vasavada, MSL Science Team, Curiosity's mission of exploration at Gale Crater, Mars. *Elements* **11**, 19–26 (2015).

17. D. Vaniman, S. Chipera, E. Rampe, T. Bristow, D. Blake, J. Meusburger, T. Peretyazhko, W. Rapin, J. Berger, D. Ming, P. Craig, N. Castle, R. T. Downs, S. Morrison, R. Hazen, R. Morris, A. Pandey, A. H. Treiman, A. Yen, C. Achilles, B. Tutolo, E. Hausrath, S. Simpson, M. Thorpe, V. Tu, D. J. Des Marais, J. Grotzinger, A. Fraeman, Gypsum on Mars: A Detailed View at Gale Crater. *Minerals* **14**, 815 (2024).

18. M. Nachon, S. M. Clegg, N. Mangold, S. Schröder, L. C. Kah, G. Dromart, A. Ollila, J. R. Johnson, D. Z. Oehler, J. C. Bridges, S. Le Mouélic, O. Forni, R. C. Wiens, R. B. Anderson, D. L. Blaney, J. F. Bell III, B. Clark, A. Cousin, M. D. Dyar, B. Ehlmann, C. Fabre, O. Gasnault, J. Grotzinger, J. Lasue, E. Lewin, R. Léveillé, S. Mc Lennan, S. Maurice, P.-Y. Meslin, W. Rapin, M. Rice, S. W. Squyres, K. Stack, D. Y. Sumner, D. Vaniman, D. Wellington, Calcium sulfate veins characterized by ChemCam/Curiosity at Gale Crater, Mars. *J. Geophys. Res.* **119**, 1991–2016 (2014).

19. R. E. Kronyak, L. C. Kah, K. S. Edgett, S. J. VanBommel, L. M. Thompson, R. C. Wiens, V. Z. Sun, M. Nachon, Mineral-filled fractures as indicators of multigenerational fluid flow in the Pahrump Hills member of the Murray Formation, Gale Crater, Mars. *Earth Space Sci.* **6**, 238–265 (2019).

20. R. C. Moeller, L. Jandura, K. Rosette, M. Robinson, J. Samuels, M. Silverman, K. Brown, E. Duffy, A. Yazzie, E. Jens, I. Brockie, L. White, Y. Goreva, T. Zorn, A. Okon, J. Lin, M. Frost, C. Collins, J. B. Williams, A. Steltzner, F. Chen, J. Biesiadecki, The Sampling and Caching Subsystem (SCS) for the Scientific Exploration of Jezero Crater by the Mars 2020 Perseverance Rover. *Space Sci. Rev.* **217**, 5 (2021).

21. A. Aubrey, H. J. Cleaves, J. H. Chalmers, A. M. Skelley, R. A. Mathies, F. J. Grunthaner, P. Ehrenfreund, J. L. Bada, Sulfate minerals and organic compounds on Mars. *Geology* **34**, 357–360 (2006).

22. R. Barnes, S. Gupta, G. Paar, K. M. Stack-Morgan, B. Horgan, L. Crumpler, M. Tebolt, G. Caravaca, S. Holm-Alwmark, T. Ortner, in *54th Lunar and Planetary Science Conference* (2023), vol. 54, pp. 2716.

23. G. Lopez-Reyes, M. Nachon, M. Veneranda, O. Beyssac, J. M. Madariaga, J. A. Manrique, E. Clavé, A. Ollila, K. Castro, S. K. Sharma, J. R. Johnson, S. Schröder, E. Cloutis, E. Dehouck, J. Huidobro, J. Martinez-Frias, F. Rull, S. Maurice, R. C. Wiens, in *54th Lunar and Planetary Science Conference*. (The Woodlands, Texas, 2023), vol. 2806, pp. 1721.

24. Y. Y. Phua, B. L. Ehlmann, S. Siljeström, A. D. Czaja, P. Beck, S. Connell, R. C. Wiens, R. S. Jakubek, R. M. E. Williams, M.-P. Zorzano, M. E. Minitti, A. C. Pascuzzo, K. P. Hand, R. Bhartia, L. C. Kah, L. Mandon, J. Razzell Hollis, E. L. Scheller, S. Sharma, A. Steele, K. Uckert, K. H. Williford, A. G. Yanchilina, Characterizing hydrated sulfates and altered phases in Jezero Crater Fan and floor geologic units With SHERLOC on Mars 2020. *J. Geophys. Res. Planets* **129**, e2023JE008251 (2024).

25. A. C. Allwood, L. A. Wade, M. C. Foote, W. T. Elam, J. A. Hurowitz, S. Battel, D. E. Dawson, R. W. Denise, E. M. Ek, M. S. Gilbert, M. E. King, C. C. Liebe, T. Parker, D. A. K. Pedersen, D. P. Randall, R. F. Sharrow, M. E. Sondheim, G. Allen, K. Arnett, M. H. Au, C. Basset, M. Benn, J. C. Bousman, D. Braun, R. J. Calvet, B. Clark, L. Cinquini, S. Conaby, H. A. Conley, S. Davidoff, J. Delaney, T. Denver, E. Diaz, G. B. Doran, J. Ervin, M. Evans, D. O. Flannery, N. Gao, J. Gross, J. Grotzinger, B. Hannah, J. T. Harris, C. M. Harris, Y. He, C. M. Heirwegh, C. Hernandez, E. Hertzberg, R. P. Hodyss, J. R. Holden, C. Hummel, M. A. Jadusingh, J. L. Jørgensen, J. H. Kawamura, A. Kitiyakara, K. Kozaczek, J. L. Lambert, P. R. Lawson, Y. Liu, T. S. Luchik, K. M. Macneal, S. N. Madsen, S. M. McLennan, P. McNally, P. L. Meras, R. E. Muller, J. Napoli, B. J. Naylor, P. Nemere, I. Ponomarev, R. M. Perez, N. Pootrakul, R. A. Romero, R. Rosas, J. Sachs, R. T. Schaefer, M. E. Schein, T. P. Setterfield, V. Singh, E. Song, M. M. Soria, P. C. Stek, N. R. Tallarida, D. R. Thompson, M. M. Tice, L. Timmermann, V.

Torossian, A. Treiman, S. Tsai, K. Uckert, J. Villalvazo, M. Wang, D. W. Wilson, S. C. Worel, P. Zamani, M. Zappe, F. Zhong, R. Zimmerman, PIXL: Planetary instrument for X-ray lithochemistry. *Space Sci. Rev.* **216**, 134 (2020).

26. M. M. Tice, J. A. Hurowitz, A. C. Allwood, M. W. M. Jones, B. J. Orenstein, S. Davidoff, A. P. Wright, D. A. K. Pedersen, J. Henneke, N. J. Tosca, K. R. Moore, B. C. Clark, S. M. McLennan, D. T. Flannery, A. Steele, A. J. Brown, M.-P. Zorzano, K. Hickman-Lewis, Y. Liu, S. J. VanBommel, M. E. Schmidt, T. V. Kizovski, A. H. Treiman, L. O'Neil, A. G. Fairén, D. L. Shuster, S. Gupta, PIXL Team, Alteration history of Séitah formation rocks inferred by PIXL X-ray fluorescence, X-ray diffraction, and multispectral imaging on Mars. *Sci. Adv.* **8**, eabp9084 (2022).

27. B. J. Orenstein, M. W. M. Jones, D. T. Flannery, A. P. Wright, S. Davidoff, M. M. Tice, L. Nothdurft, A. C. Allwood, In-situ mapping of monocrystalline regions on Mars. *Icarus* **420**, 116202 (2024).

28. C. E. Schrank, M. W. M. Jones, D. L. Howard, A. Berger, M. Herwegh, Micro-scale structural and chemical characterisation of deformed rocks with simultaneous in-situ synchrotron x-ray fluorescence and backscatter diffraction mapping. *Chem. Geol.* **645**, 121886 (2024).

29. A. Schwartz. (Springer, 2009).

30. G. Hansford, A prototype handheld x-ray diffraction instrument. *J. Appl. Crystallogr.* **51**, 1571–1585 (2018).

31. H. J. Kirkwood, M. D. de Jonge, O. Muránsky, F. Hofmann, D. L. Howard, C. G. Ryan, G. A. van Riessen, M. R. Rowles, A. M. Paradowska, B. Abbey, Simultaneous x-ray diffraction, crystallography, and fluorescence mapping using the Maia detector. *Acta Mater.* **144**, 1–10 (2018).

32. V. K. Gupta, S. R. Agnew, Indexation and misorientation analysis of low-quality Laue diffraction patterns. *J. Appl. Crystallogr.* **42**, 116–124 (2009).

33. F. C. Hawthorne, R. B. Ferguson, Anhydrous sulphates; II, Refinement of the crystal structure of anhydrite. *Can. Mineral.* **13**, 289–292 (1975).

34. J. C. A. Boeyens, V. V. H. Ichharam, Redetermination of the crystal structure of calcium sulphate dihydrate, $CaSO_4$ $2H_2O$. *Z. Kristallogr. New Cryst.Struct.* **217**, 9–10 (2002).

35. C. Bezou, A. Nonat, J.-C. Mutin, A. N. Christensen, M. S. Lehmann, Investigation of the crystal structure of γ-$CaSO_4$, $CaSO_4$ 0.5 H_2O, and $CaSO_4$ 0.6 H_2O by powder diffraction methods. *J. Solid State Chem.* **117**, 165–176 (1995).

36. M.-P. Zorzano, G. Martínez, J. Polkko, L. K. Tamppari, C. Newman, H. Savijärvi, Y. Goreva, D. Viúdez-Moreiras, T. Bertrand, M. Smith, E. M. Hausrath, S. Siljeström, K. Benison, T. Bosak, A. D. Czaja, V. Debaille, C. D. K. Herd, L. Mayhew, M. A. Sephton, D. Shuster, J. I. Simon, B. Weiss, N. Randazzo, L. Mandon, A. Brown, M. H. Hecht, J. Martínez-Frías, Present-day thermal and water activity environment of the Mars Sample Return collection. *Sci. Rep.* **14**, 7175 (2024).

37. R. C. Murray, Origin and diagenesis of gypsum and anhydrite. *J. Sediment. Petrol.* **34**, 512–523 (1964).

38. J. K. Warren, *Evaporites* (Springer Cham, ed. 2, 2016), pp. 1813.

39. D. Freyer, W. Voigt, Crystallization and phase stability of $CaSO_4$ and $CaSO_4$–based salts. *Monatshefte für Chemie* **134**, 693–719 (2003).

40. P. Launeau, C. J. Archanjo, D. Picard, L. Arbaret, P.-Y. Robin, Two- and three-dimensional shape fabric analysis by the intercept method in grey levels. *Tectonophysics* **492**, 230–239 (2010).

41. J. W. Cosgrove, S. G. Banham, S. Gupta, R. Barnes, The origin of the fracture networks in the mudstones of Gale Crater Mars; their implications regarding the state of stress and fluid pressure during their formation and the depth to which they were buried. *J. Geophys. Res. Planets* **127**, e2022JE007313 (2022).

42. D. A. Ferrill, J. Winterle, G. Wittmeyer, D. Sims, S. Colton, A. Armstrong, A. P. Morris, Stressed rock strains groundwater at Yucca Mountain, Nevada. *GSA Today* **9**, 1–8 (1999).

43. J. C. Jaeger, N. G. W. Cook, R. W. Zimmerman, *Fundamentals of Rock Mechanics* (Blackwell Pub., ed. 4, 2007).

44. B. De Toffoli, N. Mangold, M. Massironi, A. Zanella, R. Pozzobon, S. Le Mouélic, J. L'Haridon, G. Cremonese, Structural analysis of sulfate vein networks in Gale crater (Mars). *J. Struct. Geol.* **137**, 104083 (2020).

45. L. R. Feret, *La Grosseur des grains des matières pulvérulentes* (Eidgen. Materialprüfungsanstalt a. d. Eidgen. Technischen Hochschule).

46. J. Heeb, D. Healy, N. E. Timms, E. Gomez-Rivas, Rapid hydration and weakening of anhydrite under stress: Implications for natural hydration in the Earth's crust and mantle. *Solid Earth* **14**, 985–1003 (2023).

47. P. D. Bons, M. A. Elburg, E. Gomez-Rivas, A review of the formation of tectonic veins and their microstructures. *J. Struct. Geol.* **43**, 33–62 (2012).

48. W. F. Brace, E. G. Bombolakis, A note on brittle crack growth in compression. *J. Geophys. Res.* **68**, 3709–3713 (1963).

49. H. Horii, S. Nemat-Nasser, Compression-induced microcrack growth in brittle solids: Axial splitting and shear failure. *J. Geophys. Res.* **90**, 3105–3125 (1985).

50. M. Veveakis, T. Poulet, A note on the instability and pattern formation of shrinkage cracks in viscoplastic soils. *Geomech. Energy Environ.* **25**, 100198 (2021).

51. K. L. Siebach, J. P. Grotzinger, L. C. Kah, K. M. Stack, M. Malin, R. Léveillé, D. Y. Sumner, Subaqueous shrinkage cracks in the Sheepbed mudstone: Implications for early fluid diagenesis, Gale crater, Mars. *J. Geophys. Res. Planets* **119**, 1597–1613 (2014).

52. C. Konstantinou, G. Biscontin, F. Logothetis, Tensile strength of artificially cemented sandstone generated via microbially induced carbonate precipitation. *Materials* **14**, 4735 (2021).

53. S. W. Squyres, R. E. Arvidson, J. F. Bell 3rd, F. Calef, B. C. Clark, B. A. Cohen, L. A. Crumpler, P. A. de Souza, W. H. Farrand, R. Gellert, J. Grant, K. E. Herkenhoff, J. A. Hurowitz, J. R. Johnson, B. L. Jolliff, A. H. Knoll, R. Li, S. M. McLennan, D. W. Ming, D. W. Mittlefehldt, T. J. Parker, G. Paulsen, M. S. Rice, S. W. Ruff, C. Schröder, A. S. Yen, K. Zacny, Ancient impact and aqueous processes at Endeavour Crater, Mars. *Science* **336**, 570–576 (2012).

54. N. J. Tosca, S. M. McLennan, Chemical divides and evaporite assemblages on Mars. *Earth Planet. Sci. Lett.* **241**, 21–31 (2006).

55. C. Quantin-Nataf, S. Alwmark, F. J. Calef, J. Lasue, K. Kinch, K. M. Stack, V. Sun, N. R. Williams, E. Dehouck, L. Mandon, N. Mangold, O. Beyssac, E. Clave, S. H. G. Walter, J. I. Simon, A. M. Annex, B. Horgan, J. W. Rice Jr., D. Shuster, B. Cohen, L. Kah, S. Sholes, B. P. Weiss, The complex exhumation history of Jezero Crater Floor Unit and its implication for Mars Sample Return. *J. Geophys. Res. Planets* **128**, e2022JE007628 (2023).

56. J. F. Bell III, J. N. Maki, S. Alwmark, B. L. Ehlmann, S. A. Fagents, J. P. Grotzinger, S. Gupta, A. Hayes, K. E. Herkenhoff, B. H. N. Horgan, J. R. Johnson, K. B. Kinch, M. T. Lemmon, M. B. Madsen, J. I. Núñez, G. Paar, M. Rice, J. W. Rice, N. Schmitz, R. Sullivan, A. Vaughan, M. J. Wolff, A. Bechtold, T. Bosak, L. E. Duflot, A. G. Fairén, B. Garczynski, R. Jaumann, M. Merusi, C. Million, E. Ravanis, D. L. Shuster, J. Simon, M. St. Clair, C. Tate, S. Walter, B. Weiss, A. M. Bailey, T. Bertrand, O. Beyssac, A. J. Brown, P. Caballo-Perucha, M. A. Caplinger, C. M. Caudill, F. Cary, E. Cisneros, E. A. Cloutis, N. Cluff, P. Corlies, K. Crawford, S. Curtis, R. Deen, D. Dixon, C. Donaldson, M. Barrington, M. Ficht, S. Fleron, M. Hansen, D. Harker, R. Howson, J. Huggett, S. Jacob, E. Jensen, O. B. Jensen, M. Jodhpurkar, J. Joseph, C. Juarez, L. C. Kah, O. Kanine, J. Kristensen, T. Kubacki, K. Lapo, A. Magee, M. Maimone, G. L. Mehall, L. Mehall, J. Mollerup, D. Viúdez-Moreiras, K. Paris, K. E. Powell, F. Preusker, J. Proton, C. Rojas, D. Sallurday, K. Saxton, E. Scheller, C. H. Seeger, M. Starr, N. Stein, N. Turenne, J. Van Beek, A. G. Winhold, R. Yingling, Geological, multispectral, and

meteorological imaging results from the Mars 2020 Perseverance rover in Jezero crater. *Sci. Adv.* **8**, eabo4856 (2022).

57. M. Cornwall, A. Hagermann, Planetary heat flow from shallow subsurface measurements: Mars. *Planet. Space Sci.* **131**, 46–59 (2016).

58. A. C. Allwood, I. W. Burch, J. M. Rouchy, M. Coleman, Morphological biosignatures in gypsum: Diverse formation processes of Messinian (∼6.0 Ma) gypsum stromatolites. *Astrobiology* **13**, 870–886 (2013).

59. E. G. Nisbet, Archaean stromatolites and the search for the earliest life. *Nature* **284**, 395–396 (1980).

60. K. J. McNamara, S. M. Awramik, Stromatolites: A key to understanding the early evolution of life. *Sci. Prog.* **76**, 345–364 (1992).

61. E. Zaikova, K. C. Benison, M. R. Mormile, S. S. Johnson, Microbial communities and their predicted metabolic functions in a desiccating acid salt lake. *Extremophiles* **22**, 367–379 (2018).

62. S. McMahon, J. Parnell, P. B. R. Reekie, Mars-Analog calcium sulfate veins record evidence of ancient subsurface life. *Astrobiology* **20**, 1212–1223 (2020).

63. A. Das, C. M. Heirwegh, N. Gao, W. T. Elam, L. A. Wade, B. C. Clark, J. A. Hurowitz, S. J. VanBommel, M. W. M. Jones, A. C. Allwood, Energy dependence of x-ray beam size produced by polycapillary x-ray optics. *X-Ray Spectrometry* **54**, 203–213(2025).

64. C. M. Heirwegh, W. T. Elam, L. P. O’Neil, K. P. Sinclair, A. Das, The focused beam x-ray fluorescence elemental quantification software package PIQUANT. *Spectrochim. Acta Part B At. Spectrosc.* **196**, 106520 (2022).

65. S. Davidoff, P. Nemere, T. Barber, R. Stonebraker, S. M. Fedell, L. Klyne, A. Galvin, A. P. Wright, J. Corkins, A. P. Wright, M. Tice, Y. Liu, J. Hurowitz, A. Allwood, *PIXLISE Spectroscopy Analysis Software: Released Versions for Published Analyses* (OSF, 2024); https://osf.io/ure2f/.

66. M. E. Schmidt, T. V. Kizovski, Y. Liu, J. D. Hernandez-Montenegro, M. M. Tice, A. H. Treiman, J. A. Hurowitz, D. A. Klevang, A. L. Knight, J. Labrie, N. J. Tosca, S. J. Van Bommel, S. Benaroya, L. S. Crumpler, B. H. N. Horgan, R. V. Morris, J. I. Simon, A. Udry, A. Yanchilina, A. C. Allwood, M. L. Cable, J. R. Christian, B. C. Clark, D. T. Flannery, C. M. Heirwegh, T. L. J. Henley, J. Henneke, M. W. M. Jones, B. J. Orenstein, C. D. K. Herd, N. Randazzo, D. Shuster, M. Wadhwa, Diverse and highly differentiated lava suite in Jezero crater, Mars: Constraints on intracrustal magmatism revealed by Mars 2020 PIXL. *Sci. Adv.* **11**, eadr2613 (2025).

67. D. Dragoi, A. Dragoi, Modeling of energy-dispersive x-ray diffraction for high-symmetry crystal orientation. *Acta Crystallographica A* **75**, 63-79 (2019).

68. C. T. Chantler, Detailed tabulation of atomic form factors, photoelectric absorption and scattering cross section, and mass attenuation coefficients in the vicinity of absorption edges in the soft x-ray (Z=30-36, Z=60-89, E=0.1 keV-10 keV), addressing convergence issues of earlier work. *J. Synchrotron. Radiat.* **8**, 1124 (2001).

69. C. M. Heirwegh, A. Das, B. P. Ganly, W. T. Elam, Y. Liu, L. A. Wade, N. Gao, in *54th Lunar and Planetary Science Conference* (2023), p. 1708.

70. B. J. Orenstein, D. T. Flannery, L. W. Casey, W. T. Elam, C. M. Heirwegh, M. W. M. Jones, A statistical approach to removing diffraction from x-ray fluorescence spectra. *Spectrochim. Acta Part B At. Spectrosc.* **200**, 106603 (2023).

71. W. Nikonow, D. Rammlmair, Risk and benefit of diffraction in energy dispersive x-ray fluorescence mapping. *Spectrochim. Acta Part B At. Spectrosc.* **125**, 120–126 (2016).

72. R. A. Fisher, Frequency distribution of the values of the correlation coefficient in samples from an indefinitely large population. *Biometrika* **10**, 507–521 (1915).

73. D. Rowenhorst, A. D. Rollett, G. S. Roher, M. Groeber, M. Jackson, P. J. Kionijnenberg, M. De Graef, Consistent representations of an conversions between 3D rotations. *Modelling Simul. Mater. Sci. Eng.* **23**, 083501 (2015).

74. D. Healy, R. E. Rizzo, D. G. Cornwell, N. J. C. Farrell, H. Watkins, N. E. Timms, E. Gomez-Rivas, M. Smith, FracPaQ: A MATLAB™ toolbox for the quantification of fracture patterns. *J. Struct. Geol.* **95**, 1–16 (2017).

75. C. A. Schneider, W. S. Rasband, K. W. Eliceiri, NIH Image to ImageJ: 25 years of image analysis. *Nat. Methods* **9**, 671–675 (2012).

76. D. Blake, D. Vaniman, C. Achilles, R. Anderson, D. Bish, T. Bristow, C. Chen, S. Chipera, J. Crisp, D. Des Marais, R. T. Downs, J. Farmer, S. Feldman, M. Fonda, M. Gailhanou, H. Ma, D. W. Ming, R. V. Morris, P. Sarrazin, E. Stolper, A. Treiman, A. Yen, Characterization and calibration of the CheMin mineralogical instrument on Mars science laboratory. *Space Sci. Rev.* **170**, 341–399 (2012.

77. A. P. Wright, P. Nemere, A. Galvin, D. H. Chau, S. Davidoff, paper presented at the Proceedings of the 28th International Conference on Intelligent User Interfaces, Sydney, NSW, Australia, 2023.

78. J. G. Robertson, Detector sampling of optical/IR spectra: How many pixels per FWHM? *Publ. Astron. Soc. Aust.* **34**, e035 (2017).

79. M. H. B. Nasseri, G. Grasselli, B. Mohanty, Fracture toughness and fracture roughness in anisotropic granitic rocks. *Rock Mech. Rock Eng.* **43**, 403–415 (2010).

80. L. Goehring, A. Nakahara, T. Dutta, S. Kitsunezaki, S. Tarafdar, "Patterns of crack networks in homogeneous media" in *Desiccation Cracks and their Patterns*, B. Chakrabarty, Ed. (2015), pp. 145–205.

81. H.-G. Machel, Fibrous gypsum and fibrous anhydrite in veins. *Sedimentology* **32**, 443–454 (1985).

82. X. Wang, C. Schrank, M. Jones, Visualizing the fibre texture of satin spar using laboratory 2D x-ray diffraction. *J. Appl. Crystallogr.* **57**, 240–247 (2024).

83. C. K. Wentworth, A scale of grade and class terms for clastic sediments. *J. Geol.* **30**, 377–392 (1922).

84. H. Gercek, Poisson's ratio values for rocks. *Int. J. Rock Mech. Min. Sci.* **44**, 1–13 (2007).

85. K. W. Lewis, S. Peters, K. Gonter, S. Morrison, N. Schmerr, A. R. Vasavada, T. Gabriel, A surface gravity traverse on Mars indicates low bedrock density at Gale crater. *Science* **363**, 535–537 (2019).

86. J. N. Maki, D. Gruel, C. McKinney, M. A. Ravine, M. Morales, D. Lee, R. Willson, D. Copley-Woods, M. Valvo, T. Goodsall, J. McGuire, R. G. Sellar, J. A. Schaffner, M. A. Caplinger, J. M. Shamah, A. E. Johnson, H. Ansari, K. Singh, T. Litwin, R. Deen, A. Culver, N. Ruoff, D. Petrizzo, D. Kessler, C. Basset, T. Estlin, F. Alibay, A. Nelessen, S. Algermissen, The Mars 2020 engineering cameras and microphone on the Perseverance Rover: A next-generation imaging system for Mars exploration. *Space Sci. Rev.* **216**, 137 (2020).

87. W. T. Elam, B. D. Ravel, J. R. Sieber, A new atomic database for x-ray spectroscopic calculations. *Radiat. Phys. Chem.* **63**, 121–128 (2002).

88. R. Bhartia, L. W. Beegle, L. DeFlores, W. Abbey, J. Razzell Hollis, K. Uckert, B. Monacelli, K. S. Edgett, M. R. Kennedy, M. Sylvia, D. Aldrich, M. Anderson, S. A. Asher, Z. Bailey, K. Boyd, A. S. Burton, M. Caffrey, M. J. Calaway, R. Calvet, B. Cameron, M. A. Caplinger, B. L. Carrier, N. Chen, A. Chen, M. J. Clark, S. Clegg, P. G. Conrad, M. Cooper, K. N. Davis, B. Ehlmann, L. Facto, M. D. Fries, D. H. Garrison, D. Gasway, F. T. Ghaemi, T. G. Graff, K. P. Hand, C. Harris, J. D. Hein, N. Heinz, H. Herzog, E. Hochberg, A. Houck, W. F. Hug, E. H. Jensen, L. C. Kah, J. Kennedy, R. Krylo, J. Lam, M. Lindeman, J. McGlown, J. Michel, E. Miller, Z. Mills, M. E. Minitti, F. Mok, J. Moore, K. H. Nealson, A. Nelson, R. Newell, B. E. Nixon, D. A. Nordman, D. Nuding, S. Orellana, M. Pauken, G. Peterson, R. Pollock, H. Quinn, C. Quinto, M. A. Ravine, R. D. Reid, J. Riendeau, A. J. Ross, J. Sackos, J. A. Schaffner, M. Schwochert, M. O Shelton, R. Simon, C. L. Smith, P. Sobron, K. Steadman, A. Steele, D. Thiessen, V. D. Tran, T. Tsai, M. Tuite, E. Tung, R. Wehbe, R. Weinberg, R. H. Weiner, R. C. Wiens, K. Williford, C. Wollonciej, Y.-H. Wu, R. A. Yingst, J. Zan, Perseverance's Scanning Habitable Environments with Raman

and Luminescence for Organics and Chemicals (SHERLOC) Investigation. *Space Sci. Rev.* **217**, 58 (2021).

89. J. F. Bell III, S. W. Squyres, K. E. Herkenhoff, J. N. Maki, H. M. Arneson, D. Brown, S. A. Collins, A. Dingizian, S. T. Elliot, E. C. Hagerott, A. G. Hayes, M. J. Johnson, J. R. Johnson, J. Joseph, K. Kinch, M. T. Lemmon, R. V. Morris, L. Scherr, M. Schwochert, M. K. Shepard, G. H. Smith, J. N. Sohl-Dickstein, R. J. Sullivan, W. T. Sullivan, M. Wadsworth, Mars Exploration Rover Athena Panoramic Camera (Pancam) investigation. *J. Geophys. Res. Planets* **108**, 8063 (2003).
